# Evaluation of Electron Density Shifts in Noncovalent Interactions

**DOI:** 10.1021/acs.jpca.1c00830

**Published:** 2021-06-01

**Authors:** Iñigo Iribarren, Goar Sánchez-Sanz, Ibon Alkorta, José Elguero, Cristina Trujillo

**Affiliations:** † Trinity Biomedical Sciences Institute, School of Chemistry, The University of Dublin, Trinity College, Dublin, Dublin 2, Ireland; ‡ Irish Centre For High-End Computing, 7 Floor, The Tower, Grand Canal Quay, Dublin 2 D02 HP83, Ireland; § Instituto de Química Médica (IQM-CSIC), Juan de la Cierva, 3, 28006 Madrid, Spain

## Abstract

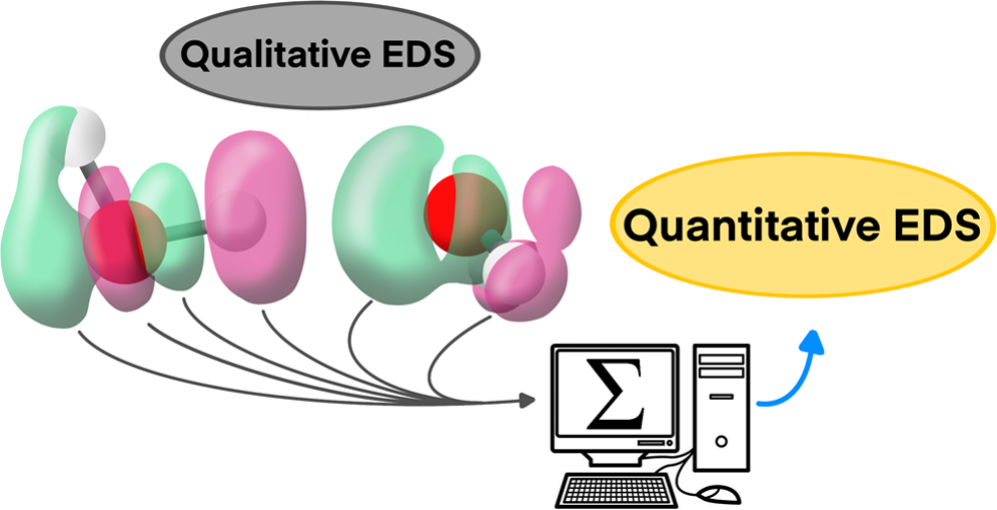

In the present paper, we report the quantitative evaluation of the electron density shift (EDS) maps within different complexes. Values associated with the total EDS maps exhibited good correlation with different quantities such as interaction energies, *E*_int_, intermolecular distances, bond critical points, and LMOEDA energy decomposition terms. Besides, EDS maps at different cutoffs were also evaluated and related with the interaction energies values. Finally, EDS maps and their corresponding values are found to correlate with *E*_int_ within systems with cooperative effects. To our knowledge, this is the first time that the EDS has been quanitatively evaluated.

## Introduction

Noncovalent interactions are of utmost importance across all domains, from chemistry to biology. The most important and best studied interaction is the hydrogen bond (HB).^1−5^ HB corresponds to the attractive interaction between a hydrogen atom (from a molecule or molecular fragment X–H, where X is more electronegative than H), and an atom within the same (intramolecular) or different (intermolecular) molecule.^6^ The second most important interaction is the halogen bond that was included by Mulliken in their theory of electron donor–acceptor complexes in the 1950s.^7^ In recent years, a number of new noncovalent interactions have been described.^8^ Their naming is derived from the periodic table column associated with the Lewis acid atoms involved in the interaction: halogen,^9−14^ chalcogen,^15−21^ pnicogen,^22−29^ tetrel,^20,30−34^ triel,^35,36^ spodium,^37^ regium,^38−42^ alkali-earth,^43,44^ and alkali^45^ bonds correspond to the interaction between an electron donor group and an atom in the Lewis acid that belongs to columns 16–11, 2 and 1 of the periodic table, respectively.

The strength of the interaction is usually assessed through the interaction energy, obtained as a difference between the energy of the complex minus the isolated monomers. It is worth noting that, in several cases, interaction energies suffer from collateral effects such as electronic repulsion, and competitive interactions that can obfuscate the estimation of the noncovalent interaction strength.

The geometrical criteria, i.e., distance between the interaction atoms, is also a well-known way to estimate the interaction between atoms. However, both aforementioned ways, can sometimes lead to ambiguous situations in which positive values of the interaction energies (repulsive) are found for stable complexes. This is mainly due to inaccurate methods, typically within exchange-correlation functionals. Examples can be found in the literature for cation–cation and anion–anion complexes.^46−49^ Also It can be found situations in which quite short distances between the interacting molecules are not associated with a bonding interaction.^50^

To provide further insight into the strength of the interactions, numerous tools and techniques have been developed in the last decades. Methods like quantum theory of atoms in molecules (QTAIM)^51−53^ and natural bond orbital (NBO)^54−56^ can provide useful information about the electron density between interacting atoms and charge transfer between monomers upon complexation. Energy decomposition schemes such as symmetry-adapted perturbation theory (SAPT),^57,58^ electron decomposition analysis (EDA),^59^ localized molecular orbital energy decomposition analysis (LMOEDA),^60^ etc., provide further information on the contribution of the different attractive and nonattractive terms to the interaction energy. In addition, the interacting quantum atom (IQA) methodology, which computes the total electronic energy as mono- and diatomic contributions, has been used to analyze the nature of chemical bonding in noncovalent interactions.^61−63^

Visual techniques that allow describing the changes upon complexation are becoming increasingly popular. These powerful techniques provide attractive graphics, which, combined with quantum chemical calculations, help the scientific community to understand those interactions. Among those techniques, one should mention noncovalent interaction index (NCI).^64^ NCI provides a very visual description of the interaction between atoms, with recent improvements toward quantification of noncovalent interactions in its latest version.^65^ On that topic, the IGMPlot technique has also recently developed and provides intrinsic bond strength index (IBSI), a numerical evaluation of the interaction between atoms.^66−68^

The electron density measures the probability of finding an electron at an infinitesimal volume of space and can be obtained by quantum mechanical models or measured using scattering methods with crystalline structures. Its analysis can be used as a tool to understand these interactions.^53,69,70^ The residual electron density maps^71^ calculated as the difference between the total electron density of a system and that generated by spherical atoms or multipolar variant are widely used in crystallography.^72,73^ More recently, it has been proposed to analyze the difference between the electron density of the complex and the isolated monomers, usually named electron density shift (EDS), as a fingerprint to characterize the interactions present in the complex. Several examples are available in the literature studying^74,75^ pnicogen interactions,^27,76^ π interactions,^77^ halogen–hydride^78^ interactions, and other noncovalent interactions.^79,80^

Electron density shift (EDS) maps have been numerously used to analyze the changes in the electron density of a certain complex upon complexation. Despite that this technique is visually very powerful and flexible, it lacks, as to our knowledge, any numerical evaluation. The electron density can be directly extracted from the wavefunction of a molecule, which is very convenient and clean in terms of evaluation and numerical manipulation.

Herein, in the present article, a thorough study of how the electron density shift map can be evaluated to provide numerical values is performed, highlighting the potential of those quantitative values to assess the strength of the interaction.

## Methods

All of the geometries were optimized at the MP2^81^/aug-cc-pVDZ^82,83^ unless otherwise stated. Cartesian coordinates of all of the complexes are presented in Table S1.

Interaction energies are defined as the difference between the energy of the complex and the energy of each monomer in the complex geometry.

The EDS maps were constructed using a three-dimensional (3D) rectangular grid of *p* points in the three directions of the space, in which the molecule is located in the center of the grid and the limit of the resulting grid was 5 Å greater than the dimensions of the molecule.

All of the calculations were carried out using Gaussian 16c01, with the cubegen tool to generate the corresponding electron density cubes from the fchk files and the cubman tool to manipulate the cubes.^84^

The calculations of the EDS total values were done using a custom-made code written in Python and plotted using the Jmol program.^85^

The topological characteristics of the electron density were studied within the quantum theory of atoms in molecules (QTAIM)^53,86^ framework with the AIMAll program.^87^

The localized molecular orbital energy decomposition analysis (LMOEDA) has been used to evaluate the importance of the different energetic term in the total interaction energy and its potential relationship with the EDS. In this method, the interaction energy is obtained as a sum of different energetic terms (eq 1)
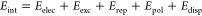
1where *E*_elect_ is the electrostatic term corresponding to the classical coulombic interaction of the occupied orbitals of one monomer with those of the other. The *E*_exc_ and *E*_rep_ terms are, respectively, the exchange and repulsive components associated with the Pauli exclusion principle, and *E*_pol_ and *E*_disp_ are the polarization and dispersion terms, respectively.^88^

## Results and Discussion

### Benchmark: Water Dimer

First of all, we will use the H_2_O···H_2_O dimer to benchmark and perform a series of analyses to calibrate the method. There are some variables to control and verify prior to evaluating the EDS values. (1) The use of full density versus frozen core, (2) the computational level, and (3) the basis set and the grid size.

The sum of all of the values of the cube grid multiplied by the increment of volume (dτ = dx*dy*d*z*) is approximately equal to the number of electrons
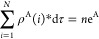
2where *ρ*^A^ is the value of the density at point *i* in the grid and *ne*^A^ is the total number of electrons in molecule A.

In an intermolecular case of a complex XY, where two monomers are interacting, X and Y, the total electron density shift, *ρ*^EDS^, is defined as
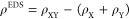
3where *ρ*_XY_, *ρ*_X_, and *ρ*_Y_ correspond to the electron density of the complex XY and the fragments X and Y. The resulting cube for the EDS complies with
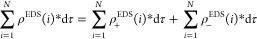
4where *ρ*_+_^EDS^ and *ρ*_–_^EDS^ correspond to the positive and negative values within the EDS cube and are associated with an increase and a decrease in the electron density upon complexation, respectively. The sum of all positive values, *ρ*_+_^EDS^, corresponds to the number of electrons accumulated
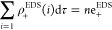
5

The same stands for *ρ*_–_^EDS^ and *n*e_–_^EDS^. For simplicity, hereafter, we will use EDS^+^ and EDS^–^ for *n*e_+_^EDS^ and *n*e_–_^EDS^, respectively. In practice, all of the density changes in the molecule should be constant, i.e., EDS^+^ – EDS^–^ should be 0.

However, there is an intrinsic error in the method due to the approximated nature of using grid when constructing and evaluating the cubes. All of the generated cubes are evaluated with the cubman utility or manually to show some inherent value errors. To evaluate the error magnitude, the absolute value of the percentage of the electron density shift with respect to the total positive (or negative) values has been defined as
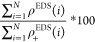
6

As observed in Table S2 for the H_2_O···H_2_O complex, the use of full density instead of frozen core produces almost identical results, with the error magnitude being very similar (0.28–0.46%). This is consistent with the idea that in noncovalent interactions, the electron transfer, i.e., the interaction, occurs only with valence electrons.

Regarding the quantum method (using aug-cc-pVDZ basis set), we evaluated the performance of DFT compared with MP2 and vice versa.^89^ The results indicate that there is a small fluctuation in the errors, with the largest for B3LYP and the smallest for MP2. This was also compared for another system (NH_3_···HF) in which DFT presents larger errors than MP2. However, in all of the cases, the errors are less than 0.6% (Table S2).

The effect of the basis set was also evaluated using aug-cc-pVDZ, jul-cc-pVTZ, aug-cc-pVTZ, and aug-cc-pVQZ. As observed (Table S2), there is no clear trend or fluctuations in the error values, so increasing the basis set does not reduce the error. As mentioned, one should keep in mind that the errors from constructing the cubes are very small (<0.6%). So, in principle, the use of a certain basis set (unless for the ones tested) does not have a significant impact on the electron density shift. This is consistent with the idea that although the total energy of the systems depends clearly on the quality of the basis set, the electron density is much less sensitive to the quality of the basis set. Hereafter, we will use MP2/aug-cc-pVDZ (unless otherwise stated).

The cube grid was also evaluated to see the impact of the size of the increment of volume (dτ) on the EDS values. As seen in Table S3, more than 30 000 grid points are more than enough to capture all of the EDS values with <0.4% error. In fact, increasing the grid points does no reduce the error. For visualization purposes, a grid with 100 input points per direction is selected.

### Total EDS Values in Noncovalent Interactions

Now let us evaluate the EDS values on a different range of noncovalent interactions. For that purpose, we select a number of complexes between a variety of molecules and ammonia (Table 1) to illustrate previously studied interactions such as hydrogen bonds (HB),^90−92^ halogen bonds (XB),^91−93^ chalcogen bonds (YB),^92,94^ pnicogen bonds (ZB),^15,92,95^ and more exotic *n*–π (also called in these particular systems orthogonal interactions)^96^ and dipole–dipole interactions.^97^ All of the molecular graphs and Cartesian coordinates are presented in Table S1.

**Table 1:** Total Positive Electrons, EDS_*T*_^+^, in e, Percentage of EDS_*T*_^+^, and Interaction Energies, in kJ/mol, at the MP2/aug-cc-pVDZ Computational Level

system	type	EDS_*T*_^+^	%EDS_*T*_^+^^a^	*E*_int_
FH···NH(CH_3_)_3_		0.2360	0.694	–78.1
FH···NH(CH_3_)_2_		0.2139	0.764	–73.4
FH···NH_2_CH_3_		0.1852	0.842	–66.6
FH···NH_3_	HB	0.1521	0.951	–58.2
O_2_NH···NH_3_		0.1625	0.625	–49.7
ClH···NH_3_		0.1889	1.181	–45.7
F_3_CH···NH_3_		0.0830	0.244	–22.7
HSH···NH_3_		0.0941	0.589	–18.3
H_2_O···H_2_O		0.0761	0.476	–22.0
H_2_S···H_2_O		0.0641	0.400	–13.4
H_2_Se···H_2_O		0.0637	0.245	–12.1
	XB			
FBr···NH_3_		0.2826	0.884	–75.9
FCl···NH_3_		0.2507	1.137	–57.9
ClBr···NH_3_		0.2426	0.758	–51.5
Br_2_···NH_3_		0.2247	0.535	–43.1
Cl_2_···NH_3_		0.1572	0.715	–29.1
O_2_NCl···NH_3_		0.1173	0.366	–16.6
F_3_CCl···NH_3_		0.0680	0.170	–13.2
HCl···NH_3_		0.0389	0.243	–4.7
	YB			
FHSe···NH_3_		0.2324	0.726	–58.3
FHS···NH_3_		0.1831	0.829	–42.9
O_2_NHSe···NH_3_		0.2005	0.477	–40
O_2_NHS···NH_3_		0.1379	0.431	–29.8
CNHSe···NH_3_		0.1097	0.323	–29.2
F_3_CHSe···NH_3_		0.1008	0.202	–22.8
CNHS···NH_3_		0.0861	0.359	–22.6
F_3_CHS···NH_3_		0.0777	0.194	–18.2
	ZB			
O_2_NH_2_As···NH_3_		0.2137	0.509	–43.2
FH_2_As···NH_3_		0.1829	0.577	–43.2
O_2_NH_2_P···NH_3_		0.1810	0.566	–35.9
FH_2_P···NH_3_		0.1557	0.708	–34.2
F_3_CH_2_As···NH_3_		0.1107	0.222	–23.1
F_3_CH_2_P···NH_3_		0.0963	0.241	–19.4
H_3_As···NH_3_		0.0573	0.221	–11.1
H_3_P···NH_3_		0.0466	0.292	–8.6
	*n*-π			
FNO_2_···NH_3_		0.0776	0.242	–23.6
FNO_2_···H_2_O		0.0763	0.238	–22.4
	μ–μ			
(PH_2_CN)_2_		0.1158	0.362	–31

a The percentage of electrons is referred to the total number on the complex.

Table 1 provides the total positive electron density shift values (EDS_T_^+^). The values of EDS_T_^+^ are relatively small if those are compared with the total number of electrons, which, in principle, is consistent with the idea of noncovalent interactions, i.e., weak interactions. It is worth remembering that EDS_T_^+^ values correspond to the total number of electrons displaced upon complexation. In fact, this is confirmed by the percentage of the electrons displaced (%EDS_T_^+^) with respect to the total number of electrons in the complex. In all of the cases, the percentage found is less than 1.2%.

**Figure 1:**
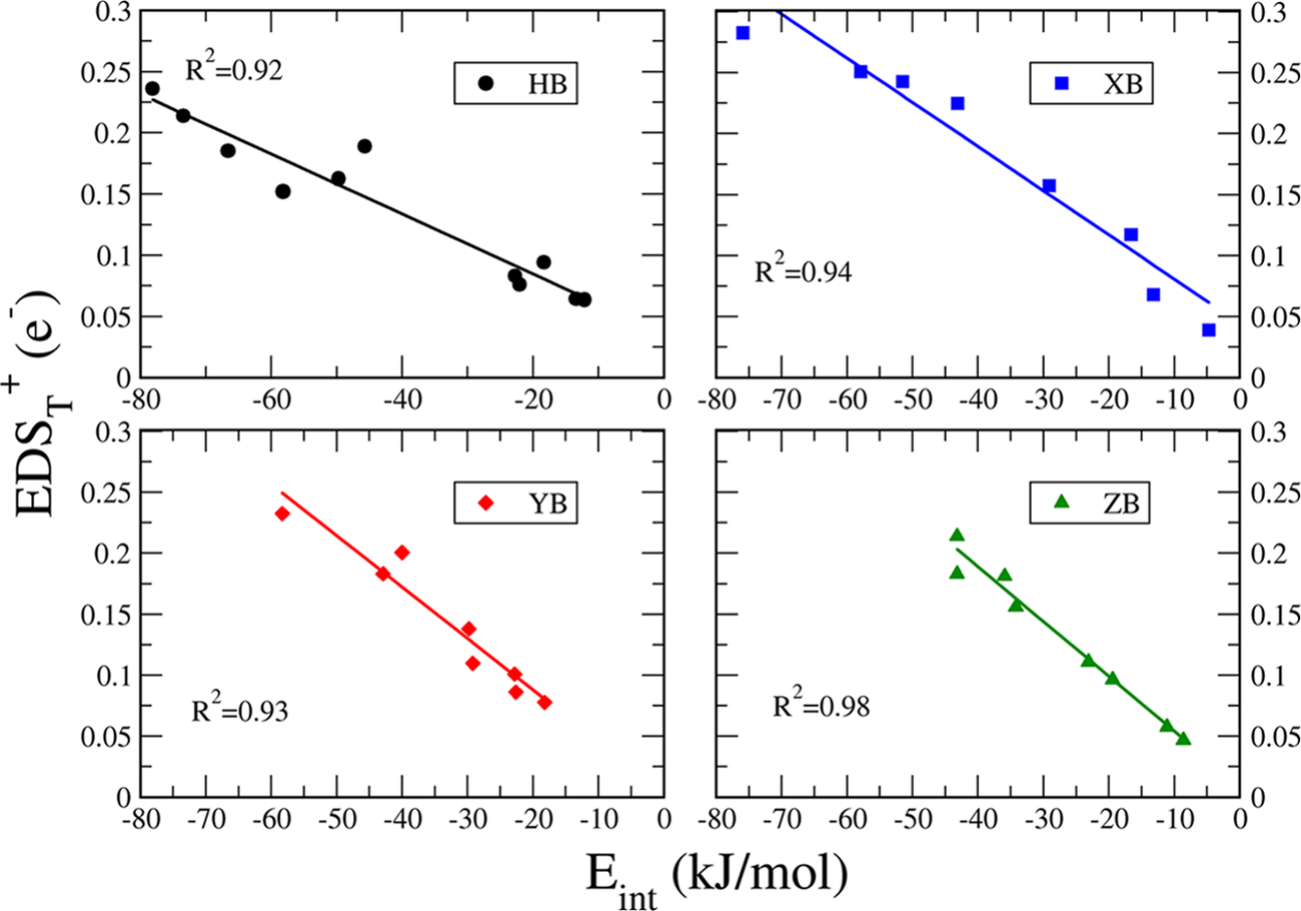
Correlations between the positive electron density values (EDS_T_^+^) and the interaction energy (*E*_int_) for each family of interactions.

In fact, a correlation between the EDS_T_^+^ values and the interaction energy (Figure 1) was found (*R*^2^ = 0.92, 0.94, 0.93, and 0.98 for HB, XB, YB, and ZB interactions, respectively).

The correlation is fair but not perfect. What is the reason behind? One should keep in mind that EDS captures not only the intermolecular electron density changes upon complexation but also the intramolecular density relocation. This can be the reason why in more polarizable molecules the EDS is greater, due to the change in the total electron density, but the interaction energy, usually dominated by charge transfer, does not correlate with EDS values.

Nevertheless, as expected, the EDS values are related with the interaction between both monomers. But are those EDS values related with other quantities?

**Figure 2:**
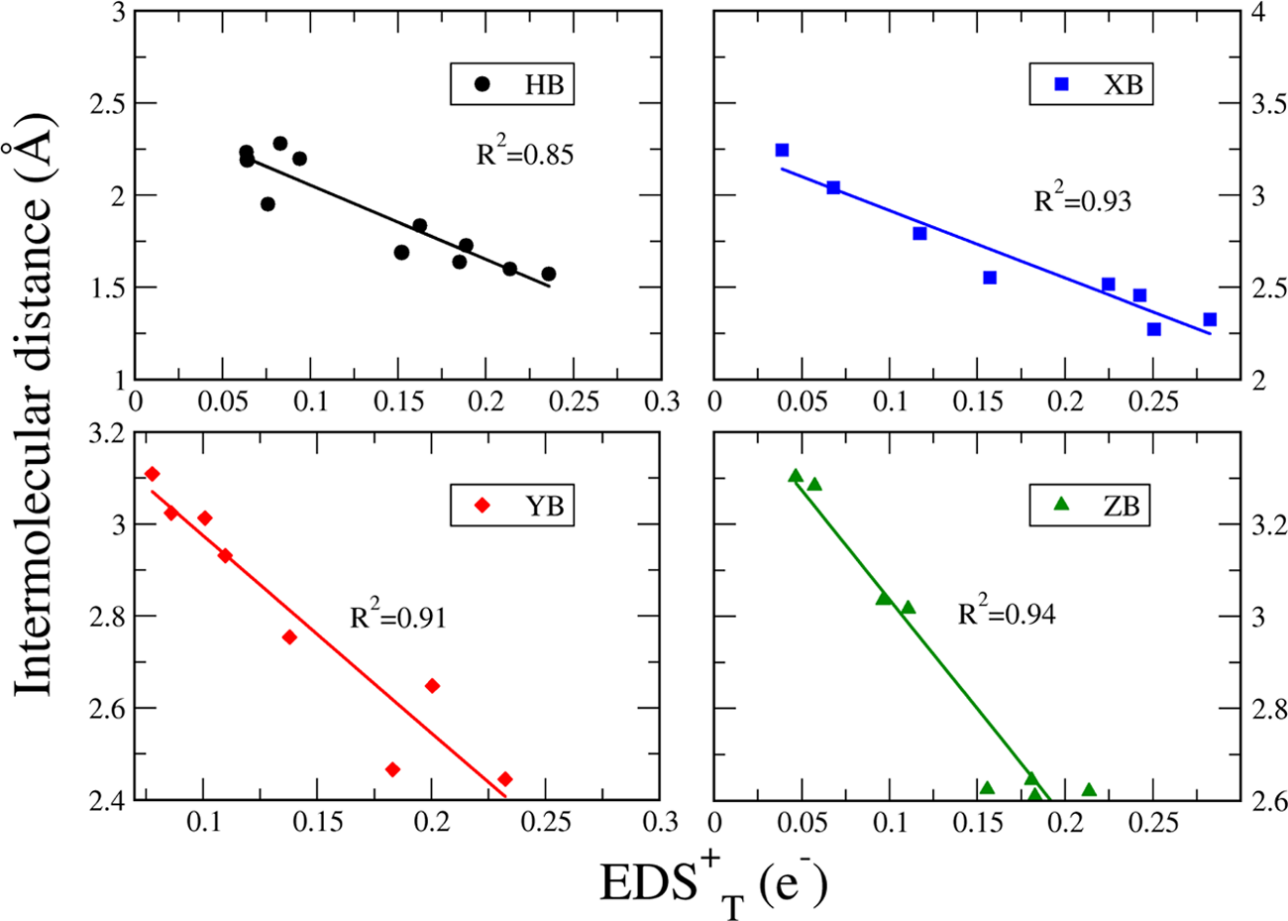
Correlation between the intermolecular distance of the interacting atoms and EDS_T_^+^ for each family of interactions studied at the MP2/aug-cc-pVDZ computational level.

**Figure 3:**
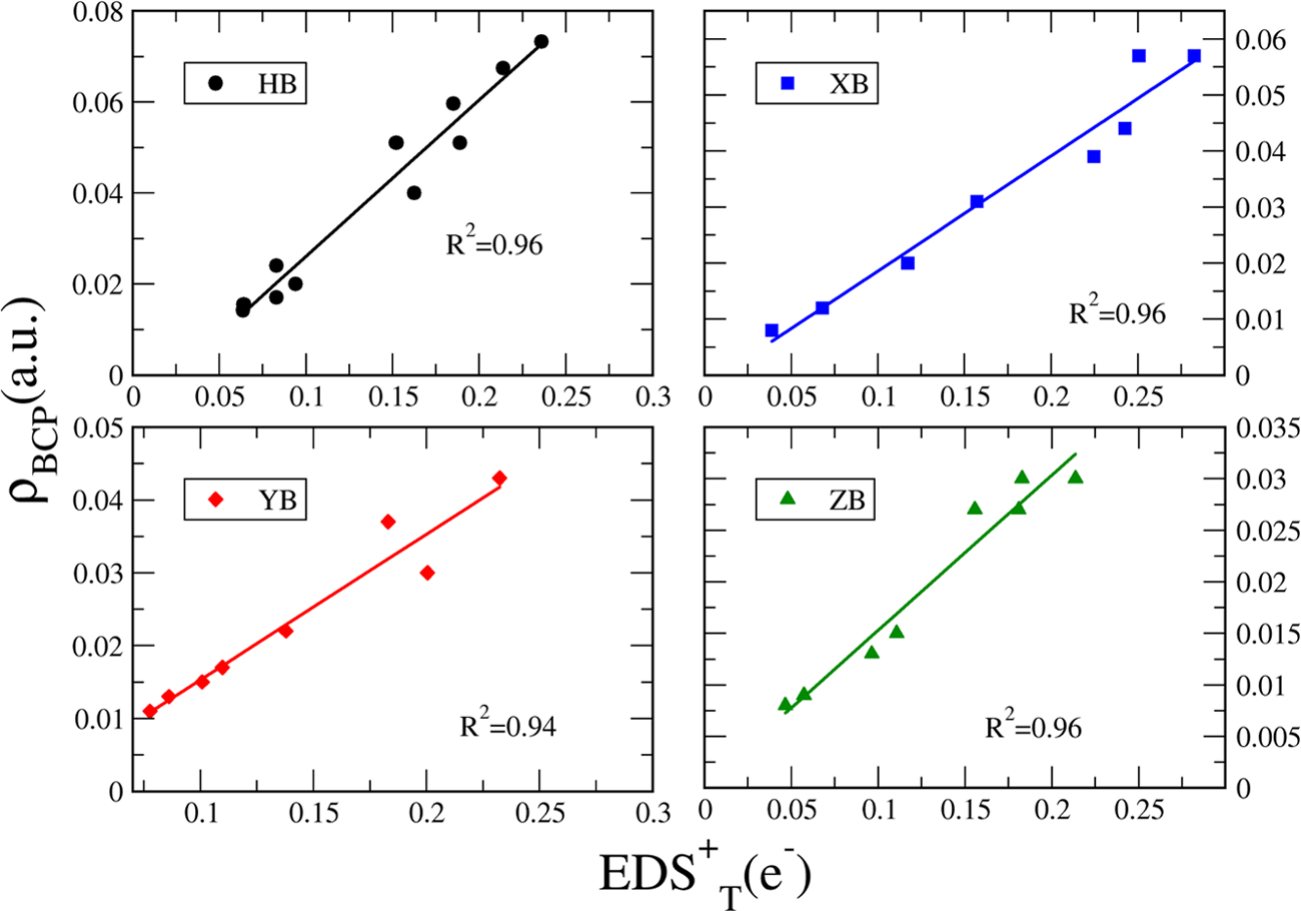
Correlation between the electron density at the BCP and EDS_T_^+^ for each family of interactions studied at the MP2/aug-cc-pVDZ computational level.

The quantum theory of atoms in molecules (QTAIM) has been used to analyze the electron density properties of the bond critical points (BCP) between the interacting atoms. Table S4 presents the intermolecular distances and the electron densities, (ρ_BCP_), at the BCP. Linear correlations were found between EDS_T_^+^ and the intermolecular distance (*R*^2^ = 0.93, 0.91, and 0.94 for XB, YB, and ZB interactions, respectively) in Figure 2, and even better correlations were found with ρ_BCP_ (*R*^2^ = 0.96, 0.96, 0.94, and 0.96 for HB, XB, YB, and ZB interactions, respectively) in Figure 3. Those correlations reinforce the relationship between the EDS and other quantities commonly used to evaluate the strength of intermolecular interactions. Also, as observed for *E*_int_, those correlations are not perfect, particularly for HBs (*R*^2^ = 0.85 for EDS_T_^+^ vs distance), since the EDS involves intra- and intermolecular electron density displacements. Still, the EDS_T_^+^ values provide a fair view of the interaction and complement the visual EDS maps as will be presented in the next section.

LMOEDA calculations were carried out to evaluate the possible correlation of the different energy interaction terms with the EDS_T_^+^ values (Table S5). In all cases, the most important term in absolute values corresponds to the repulsion energy. With respect to the attractive terms, the most important one is the exchange, which represents between 40.6 and 51.1% of the sum of all of the attractive terms followed by the electrostatic one (between 23.4 and 40.9% of the attractive terms). The two remaining terms, polarization and dispersion, show contributions between 8.5 and 19.6% and 0.4 and 15.7%, respectively.

With the exception of the dispersion term, the remaining energetic terms show acceptable correlations with the EDS_T_^+^. Second-order polynomial correlations were found between EDS_T_^+^ and the three more important attractive terms for all of the complexes considered (Figure S1). The best statistical results were obtained when the *E*_exc_ + *E*_pol_ terms were compared with EDS_T_^+^. The *E*_exc_ term takes into account the bonding character formed in the intermolecular region, while the polarization term (*E*_pol_) accounts for the electronic deformation within each molecule. The combination of both terms, *E*_exc_ + *E*_pol_, accounts for the electronic displacement in the binary complexes studied.

### EDS Values and Isosurface Cutoffs

**Figure 4:**
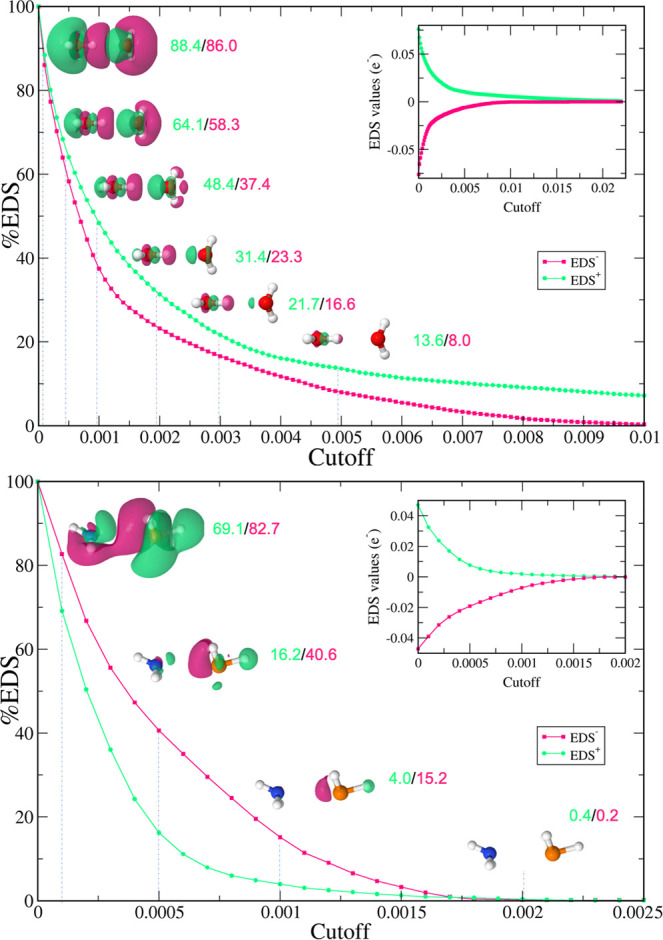
Evolution of the percentage of EDS inside the cutoff (a.u.) and EDS values (in-plots) with respect to the cutoff for H_2_O···H_2_O (top) and PH_3_···NH_3_ (bottom) systems. Green and magenta represent positive and negative values of the EDS, respectively.

Although the total EDS_T_(+ or −) values are useful to evaluate the electron density displaced upon complexation, the EDS maps at different isovalues are usually presented to illustrate those changes. In fact, there is not an established value to plot the EDS maps, and a cutoff of 0.001 a.u. is usually selected. The evolution of the EDS cutoff (EDS_c_) with the %EDS (% w.r.t. to the EDS_T_^+^) was analyzed for three different systems: H_2_O···H_2_O, FBr···NH_3_, and PH_3_···NH_3_. In Figures 4 and S2, the evolution of the EDS with respect to the cutoff was plotted.

As observed, in the inset plot, both EDS_c_^+^ and EDS_c_^–^ values converged rapidly with the cutoff. However, more illustrative is the percentage of the EDS inside a certain cutoff, %EDS_c_. A relatively strong interaction like H_2_O···H_2_O (Figure 4, top) presents a smooth decay, for example, at a 0.0005 a.u. cutoff, the %EDS_c_^+^ and %EDS_c_^–^ are 64.1/58.3, and when the cutoff is 0.001 a.u. those values become 48.4/37.4. But even at larger cutoff values (e.g., 0.005), EDS_c_ can be observed (13.8/8.0). This slow decay is even more evident for complexes with stronger interaction energies (FBr···NH_3_, Figure S2). On the contrary, a weaker interaction like PH_3_···NH_3_ (Figure 4, bottom) exhibits a rapid decay in the EDS_*c*_ and %EDS_c_ values, from cutoff 0.0001 (69.1/82.7) to 0.001 (4.0/15.2), which makes these types of EDS only visualized at very small cutoff values. This is also applicable to very weak systems, particularly those in which the dispersion force is dominant.^98,99^ We have explored additional complexes (ClNO_2_)_2_ and (FNO_2_)_2_ in which SAPT-DFT calculations showed that the dispersion force was the most attractive term.^98^ As observed in Figure S3, those systems present an abrupt decay of the EDS value with the cutoff value, but still it can be traced using EDS maps.

**Figure 5:**
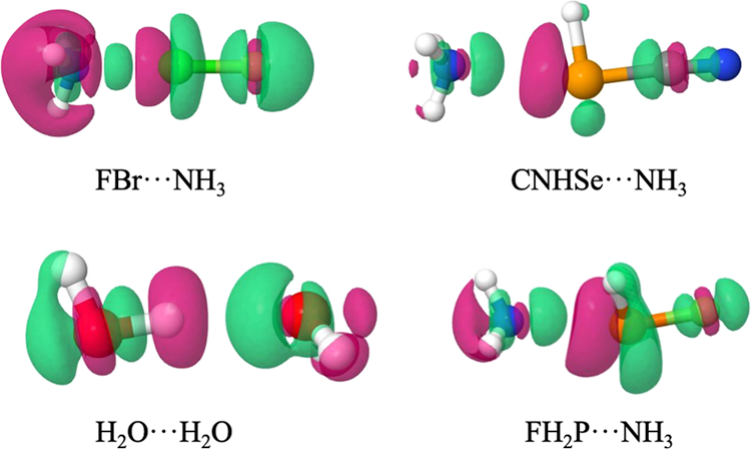
Representative EDS maps at the 0.001 a.u. isovalue at the MP2/aug-cc-pVDZ computational level. Green and magenta represent positive and negative values of the EDS, respectively.

In view of the above values, it seems reasonable to plot EDS at the 0.001 isosurface, EDS_0.001_, with the exception of those very weak interactions. All of the EDS maps for each system studied are depicted in Figure S4, and some representative examples are given in Figure 5.

The EDS_0.001_^+^ (and EDS_0.001_^–^) values and maps for each system considered can be also found in Figure S4, and in line with the total EDS_T_^+^ values, they correlate with the interaction energy (*R*^2^ = 0.87, 0.975, 0.95, and 0.98 for HB, XB, YB, and ZB interaction, respectively, Figure S5). Therefore, as observed, there is an increase and decrease in area in between both interacting atoms, which characterized the electron density transfer. In addition, it is also observed the electron density changes within the intramolecular moieties in both monomers accounting for the electron density relocalization upon complexation. In summary, the EDS maps concomitantly with their corresponding values provide a qualitative and quantitative tool to analyze a wide variety of interactions.

### EDS in Cooperative Systems

**Figure 6:**
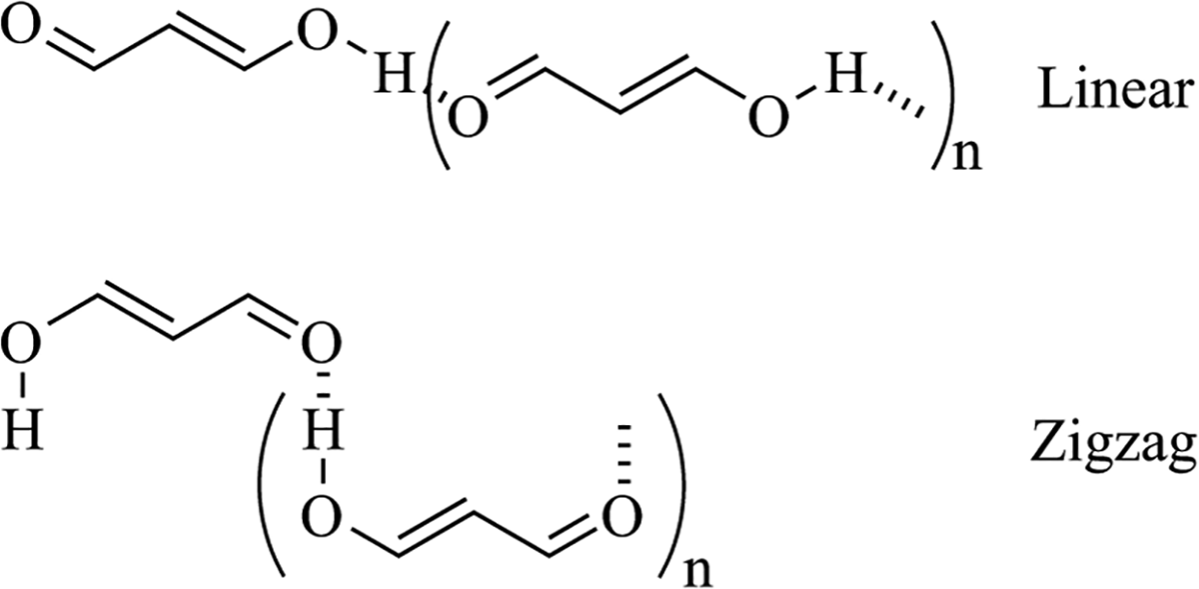
Schematic representation of the system to be studied, both linear (L_*n*_) and zigzag (Z_*n*_) conformations.

**Figure 7:**
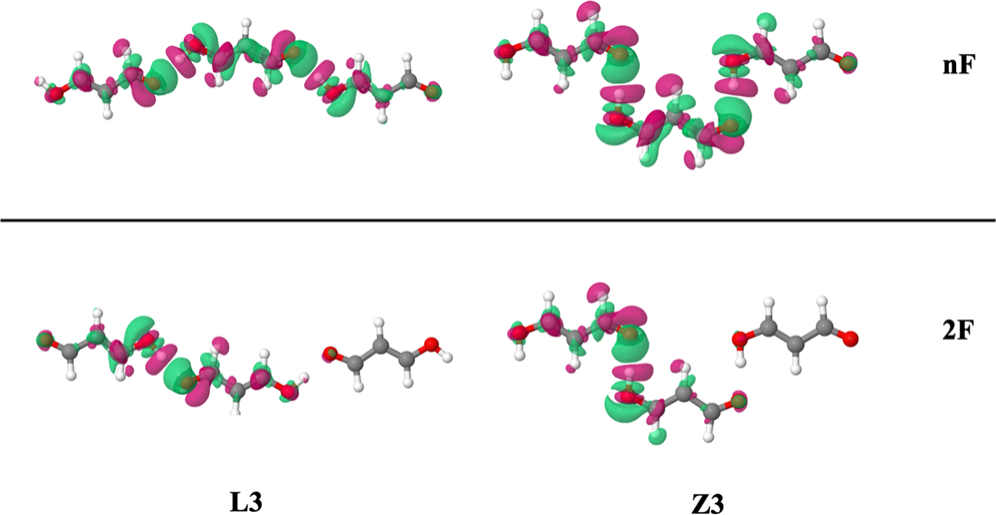
EDS maps at the 0.001 a.u. cutoff for L_3_ and Z_3_ using both *n*F and 2F fragmentation schemes. Green and magenta represent positive and negative values for the EDS, respectively.

EDS maps and the EDS^+^ (and EDS^–^) values are shown to be very illustrative to analyze intermolecular interactions. We were curious about their application to larger systems and particularly other noncovalent scenarios, for example, how the EDS maps vary with the cooperativity. Following our previous works in resonance-assisted hydrogen bonding and cooperativity,^100^ we select a system, malonaldehyde enol and its catemers, up to six monomers, to study the evolution of the EDS to infer whether this technique is able to track the changes of the electron density with increased number of monomers. Also, two different configurations have been considered. For simplicity, we only selected the unsaturated systems (compound **18** from ref (100)) (Figure 6). Two types of fragmentation to analyze the EDS values were defined
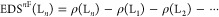
7
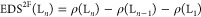
8where the first scheme EDS^*n*F^(L*_n_*) corresponds to the EDS of the linear catemer (L*_n_*) in which all of the HBs are evaluated simultaneously, i.e., the EDS is the density of the complex, ρ(L*_n_*), minus the density of each of the fragments, while the second one, EDS^2F^(L*_n_*), corresponds only to the evaluation of the most external HB, i.e., the EDS is the density of the complex minus the density of the two fragments: the one from the first monomer and the one corresponding to the rest of the complex. Table 2 presents the values of the EDS_T_^+^, EDS^+^ at the 0.001 cutoff, and the corresponding interaction energy for all of the cooperative systems. Some representative systems are plotted in Figure 7.

**Table 2:** Total (EDS_*T*_^+^) and at 0.001 Cutoff (EDS_0.001_^+^), Positive Electron Density Shift, in e^–^, and Interaction Energies, in kJ/mol, for the Two Fragmentation Schemes at the MP2/aug-cc-pVDZ Computational Level

	2F			*n*F		
sys.	EDS_T_^+^	EDS_0.001_^+^	*E*_int_	EDS_T_^+^	EDS_0.001_^+^	*E*_int_
L_2_	0.211	0.108	–52.9	0.211	0.108	–52.9
L_3_	0.257	0.128	–63.5	0.448	0.273	–117.9
L_4_	0.273	0.136	–67.4	0.702	0.463	–188.5
L_5_	0.280	0.138	–69.1	0.963	0.662	–261.9
L_6_	0.284	0.141	–69.9	1.239	0.875	–336.8
Z_2_	0.213	0.110	–52.2	0.213	0.110	–52.2
Z_3_	0.260	0.128	–59.4	0.442	0.259	–112.9
Z_4_	0.273	0.131	–63.3	0.688	0.435	–178.5
Z_5_	0.286	0.139	–64.8	0.942	0.623	–246.9
Z_6_	0.290	0.141	–65.8	1.200	0.818	–316.8

**Figure 8:**
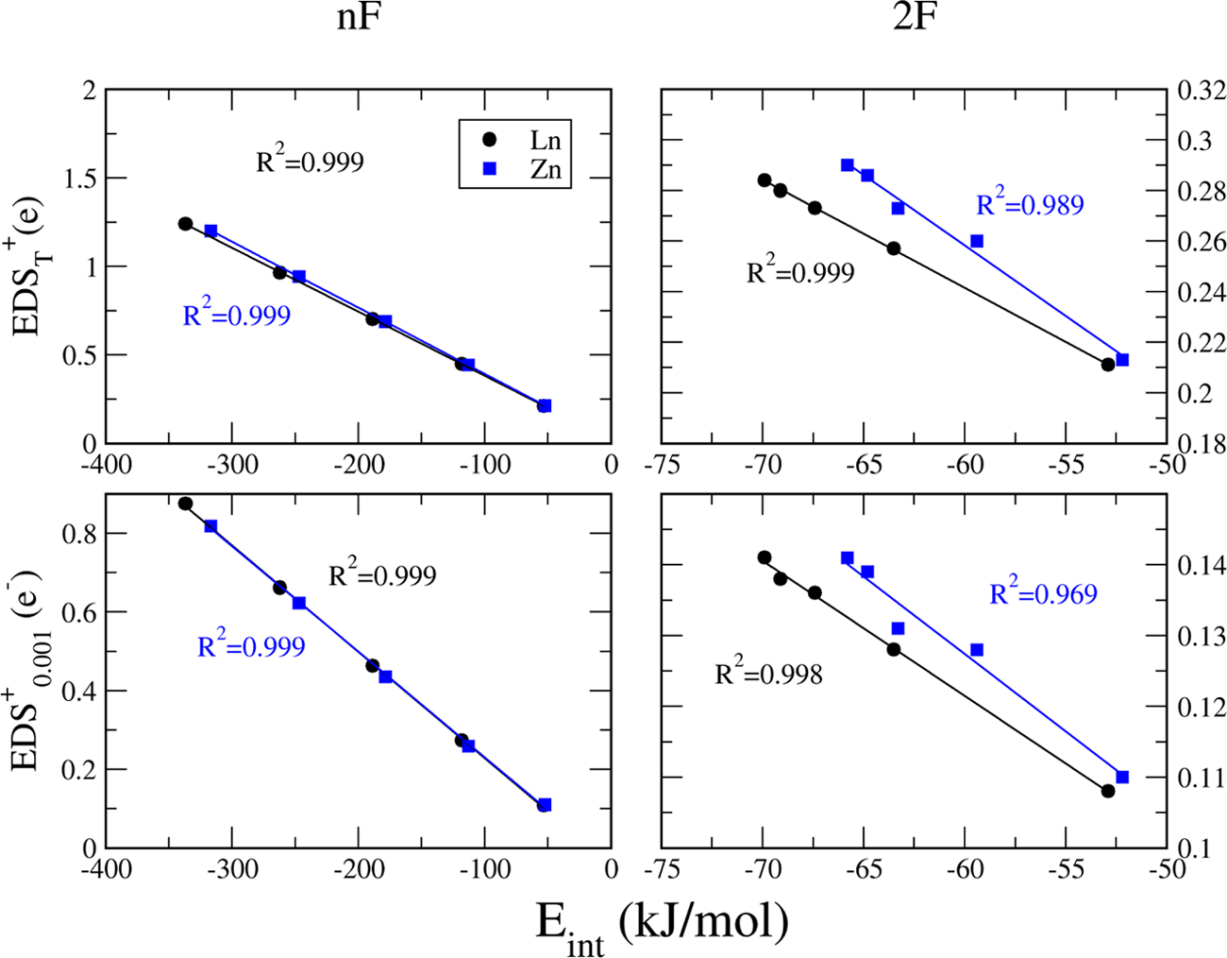
Correlation between EDS_0.001_^+^, EDS_T_^+^, and the interaction energies for each L_*n*_ and Z_*n*_ cooperative system studied at the MP2/aug-cc-pVDZ Computational Level.

As observed for the multiple fragmentation scheme (*n*F) starting from L_2_ or Z_2_ (i.e., only one HB), the addition of a subsequent monomer (L_3_ and Z_3_) increases the interaction energy more than twice. The same happens for L_4_ (Z_4_), L_5_ (Z_5_), and L_6_ (Z_6_). This is an indication of the cooperative effect and was previously reported by us. The values of both EDS_T_^+^ and EDS_0.001_^+^ perfectly correlate with the interaction energies in both L*_n_* and Z*_n_* cases, indicating that those values can capture the cooperative effect associated with the addition of monomers (Figure 8).

Focusing on just the terminal HB (Figures 7, EDS_T_^+^ and EDS_0.001_^+^ values present good linear correlation with the interaction energy associated with that particular HB (Figure 8). Again, that reinforces the viability of using EDS values to support interaction strengths.

## Conclusions

The analysis of the electron density shift has shown that EDS maps concomitantly with their corresponding values are a qualitative and quantitative tool to analyze a wide variety of interactions. Values of the total EDS have shown to present good correlation with interaction energies, electron densities at the bond critical points, and intermolecular distances. Furthermore, values at different cutoffs can present an overview of the electron density shift decay in both strong and weak interactions. Particularly, EDS maps at 0.001 a.u. have been shown to be useful to illustrate changes in noncovalent interactions, and we proved that the corresponding values also correlate with the interaction energies. Finally, EDS maps are able to capture the cooperative nature of intermolecular chains of HBs independently of the fragmentation scheme chosen.

The quantitative evaluation of EDS maps will be a useful tool to analyze noncovalent interactions henceforth.

## Supporting Information Available

Molecular graphs, Cartesian coordinates, benchmark data, electron density and intermolecular distances, LMOEDA energy terms, correlations, and electron density shift maps for all complexes studied (PDF)
