# Supplementary material for: Evaluation of Electron Density Shifts in Noncovalent Interactions
Source: J Phys Chem A. 2021 Jun 1;125(22):4741–9. doi: 10.1021/acs.jpca.1c00830 (PMC8279648; doi:10.1021/acs.jpca.1c00830)

Supplementary Information

Evaluation of Electron Density Shifts in Non-covalent Interactions

Iñigo Iribarren,† Goar Sánchez-Sanz,‡ Ibon Alkorta,¶ José Elguero,¶ and Cristina Trujillo*,†

†Trinity Biomedical Sciences Institute, School of Chemistry, The University of Dublin,Trinity College, Dublin, Dublin 2, Ireland

‡Irish Centre For High-End Computing, 7 Floor, The Tower, Grand Canal Quay, Dublin 2,d02hp83, Ireland

¶Instituto de Química Médica (IQM-CSIC), Juan de la Cierva, 3, 28006-Madrid, Spain
E-mail: trujillc@tcd.ie

Table S1. Molecular graphs and Cartesian coordinates for all the complexes studied at the MP2/aug-cc-pVDZ computational level.

Table S2. Number of Electrons, EDS total values (ρ^{EDS}), EDS positive (ρ_{+}^{EDS}) and negative values, and errors for $H_2O\cdots H_2O$ and $NH_3\cdots HF$ bonded by hydrogen bonds at different methods and basis sets.

Table S3. Input points, number of points on each direction of the grid, total number of points in the grid, increment of volume ($Bohr^3$) and electron density values (e^-) and error at the MP2/aug-cc-pVDZ for $H_2O\cdots H_2O$ system.

Table S4. Intermolecular distance, electron density (ρ_{BCP}), Laplacian ($\nabla^2\rho_{BCP}$) and total energy density (H_{BCP}) at the bond critical point for all the systems studied at the MP2/aug-cc-pVDZ for $H_2O\cdots H_2O$ system.

Table S5. LMOEDA energy components, kJ mol^{-1} for all the systems studied.

Figure S1. Correlation between the LMOEDA and the EDS^+ for each family of interactions studied at the MP2/aug-cc-pVDZ computational level

Figure S2. Evolution of the percentage of EDS inside the cut-off and EDS values (in-plots) with respect to the cut-off for $FBr\cdots NH_3$ system.

Figure S3. Electron density shift maps and $EDS^{+/-}$ values at the 0.001 a.u. isovalue for all the complexes studied at the MP2/aug-cc-pVDZ computational level.

Figure S4. Correlation between the interaction energy and the EDS^+ at the 0.001 au cut-off for each family of interactions studied at the MP2/aug-cc-pVDZ computational level.

Table S1. Molecular graphs and Cartesian coordinates for all the complexes studied at the MP2/aug-cc-pVDZ computational level.

Compound	Coordinates			
$Br_2\cdots NH_3$				
Imaginary frequencies=0	N	3.233935	-0.000288	0.000351
SCF energy=-5200.9685645	H	3.599558	-0.805154	0.509016
MP2 Energy=-5201.4575534	H	3.600464	0.842126	0.443315
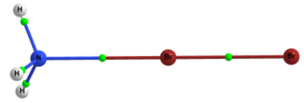	H	3.601153	-0.038417	-0.950399
	Br	-1.672586	-0.000102	0.000127
	Br	0.717194	0.000201	-0.000253
$ClBr\cdots NH_3$				
Imaginary frequencies=0	N	2.660902	0.000122	0.000047
SCF energy=-3088.0742779	H	3.023393	0.433376	0.849169
MP2 Energy=-3088.5726468	H	3.024066	0.519081	-0.799270
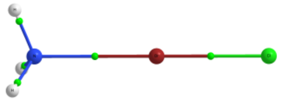	H	3.024570	-0.951405	-0.049270
	Br	0.204786	-0.000109	-0.000053
	Cl	-2.050933	0.000112	0.000052
$Cl_2\cdots NH_3$				
Imaginary frequencies=0	N	2.752693	0.000146	0.000098
SCF energy=-975.1713814	H	3.125452	0.459405	0.830824
MP2 Energy=-975.6750228	H	3.126419	0.490355	-0.812304
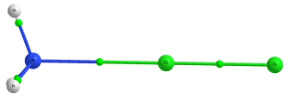	H	3.127248	-0.948201	-0.017540
	Cl	-1.885687	0.000167	0.000107
	Cl	0.200513	-0.000319	-0.000205
$ClH\cdots NH_3$				
Imaginary frequencies=0	H	0.001073	0.158331	0.000000
SCF energy=-516.3057791	Cl	0.000095	-1.184709	-0.000000
MP2 Energy=-516.6724462	N	0.000095	1.883991	0.000000
	H	0.945363	2.267639	0.000000
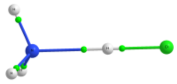	H	-0.474359	2.263074	0.819703
	H	-0.474359	2.263074	-0.819703
$CNHSe\cdots NH_3$				
Imaginary frequencies=0	N	-2.946551	0.158114	0.000302
SCF energy=-2548.9054971	H	-3.364140	0.608591	-0.815008
MP2 Energy=-2549.5520696	H	-3.362978	0.603938	0.818760
	H	-3.290931	-0.802854	-0.002205
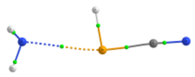	C	1.819650	0.101414	0.000167
	N	3.008466	0.180825	0.000390
	Se	-0.031018	-0.138082	-0.000173
	H	-0.278650	1.304064	-0.001521
$CNHS\cdots NH_3$				
Imaginary frequencies=1	N	-2.968502	0.141949	0.000019
SCF energy=-546.6260364	H	-3.426145	0.555897	-0.813122
MP2 Energy=-547.2687853	H	-3.423834	0.548081	0.818379
	H	-3.230382	-0.844690	-0.004349
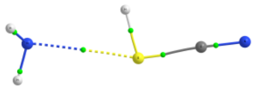	C	1.725004	0.054030	0.000009
	N	2.907999	0.191585	0.000108
	S	0.029730	-0.248215	-0.000076
	H	-0.321826	1.053222	-0.000633
$F_3CH_2As\cdots NH_3$				
Imaginary frequencies=0	N	3.591501	0.240763	0.000011
SCF energy=-2627.7522095	H	3.970993	1.188398	0.000214
MP2 Energy=-2628.8465879	H	3.990333	-0.226192	-0.815302
	H	3.990395	-0.226566	0.815078
	H	0.823632	0.764903	-1.107351
	H	0.823618	0.764701	1.107518

C	-1.367089	0.079088	0.000003
F	-1.747037	1.391433	0.000135
F	-1.951217	-0.491915	-1.098373
F	-1.951241	-0.492142	1.098248
As	0.615409	-0.245197	-0.000010

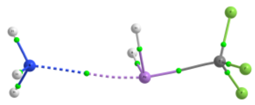

$F_3CCl\cdots NH_3$
Imaginary frequencies=0
SCF energy=-851.9323037
MP2 Energy=-853.0274931

Cl	-0.653504	-0.000659	-0.000145
N	-3.693142	0.000238	-0.000039
H	-4.084264	0.777904	0.532535
H	-4.085342	-0.849133	0.407543
H	-4.086143	0.072627	-0.939019
C	1.097477	0.000087	0.000001
F	1.578887	-0.096570	1.255486
F	1.579484	-1.038570	-0.711522
F	1.578568	1.135986	-0.543778

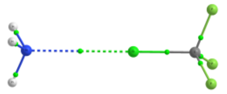

$F_3CH\cdots NH_3$
Imaginary frequencies=0
SCF energy=-393.0229810
MP2 Energy=-393.9751497

N	2.858085	-0.000228	0.000121
H	3.252434	0.309558	-0.888522
H	3.252418	-0.924690	0.176289
H	3.252132	0.614612	0.712767
C	-0.518620	-0.000092	0.000057
H	0.577967	-0.000558	0.000356
F	-1.007887	1.243992	-0.237019
F	-1.009034	-0.416617	1.195602
F	-1.008615	-0.827017	-0.958814

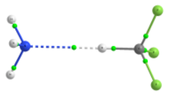

$F_3CH_2P\cdots NH_3$
Imaginary frequencies=0
SCF energy=-734.3252398
MP2 Energy=-735.4053322

P	0.727574	0.000278	-0.308307
H	0.965084	1.046577	0.623965
H	0.965166	-1.047394	0.622399
N	3.734108	-0.000092	0.103275
H	4.220106	-0.001110	1.001001
H	4.078508	0.815090	-0.405340
H	4.078287	-0.814288	-0.407067
C	-1.136042	-0.000021	0.021872
F	-1.498742	-0.001043	1.339021
F	-1.725279	1.098869	-0.538918
F	-1.725231	-1.098079	-0.540603

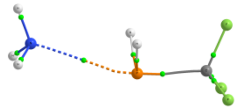

$F_3CHSe\cdots NH_3$
Imaginary frequencies=1
SCF energy=-2792.8188320
MP2 Energy=-2793.9166419

N	-3.583436	0.189035	0.000139
H	-3.920142	-0.322129	0.816803
H	-3.920150	-0.322872	-0.816057
H	-4.077986	1.082002	-0.000267
H	-0.810940	1.266290	0.000038
C	1.344734	0.060293	-0.000048
F	1.805725	0.733362	-1.088485
F	1.805804	0.733323	1.088378
F	1.933563	-1.165707	-0.000091
Se	-0.592970	-0.179326	0.000017

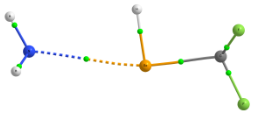

$F_3CHS\cdots NH_3$
Imaginary frequencies=0
SCF energy=-156.2537819
MP2 Energy=-156.6816372

N	1.218552	0.000055	0.000014
H	1.602551	-0.470444	0.819477
H	1.602310	-0.474893	-0.816996
H	1.603475	0.944600	-0.002612
F	-1.429809	0.000013	0.000010
H	-0.469918	0.000234	-0.000052

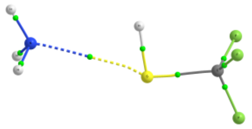

$FH_2As\cdots NH_3$
Imaginary frequencies=0
SCF energy=-2390.9983558
MP2 Energy=-2391.5586969

N	1.150701	-2.093354	0.000000
H	0.532223	-2.905309	0.000000
H	1.755337	-2.185415	0.817013
H	1.755337	-2.185415	-0.817013
H	-0.732702	-0.496408	1.103655
H	-0.732702	-0.496408	-1.103655
F	-1.181377	1.634929	0.000000
As	0.000000	0.248729	0.000000

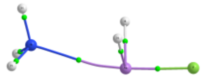

$FBr\cdots NH_3$
Imaginary frequencies=0

N	2.184553	-0.000017	-0.000031

SCF energy=-2727.9832585
MP2 Energy=-2728.5502962

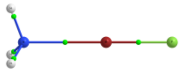

H	2.541997	-0.387344	-0.873049
H	2.542563	-0.562408	0.771651
H	2.542570	0.949497	0.100909
Br	-0.140437	0.000022	0.000041
F	-2.000411	-0.000044	-0.000079

FCl···NH_3
Imaginary frequencies=0
SCF energy=-615.0677510
MP2 Energy=-615.6435216

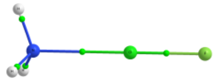

N	-2.047126	-0.000067	0.000107
H	-2.402178	0.363006	-0.883825
H	-2.402813	0.583582	0.756456
H	-2.401959	-0.947237	0.127338
Cl	0.222447	0.000139	-0.000112
F	1.972803	-0.000139	0.000132

FH···NH_3
Imaginary frequencies=0
SCF energy=-156.2537853
MP2 Energy=-156.6816374

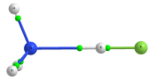

F	-0.000017	1.429981	0.000000
N	-0.000017	-1.218934	0.000000
H	-0.945160	-1.602171	0.000000
H	0.472490	-1.602559	0.818436
H	0.472490	-1.602559	-0.818436
H	0.000459	0.469999	0.000000

FH_2P···NH_3
Imaginary frequencies=0
SCF energy=-497.5674191
MP2 Energy=-498.1130322

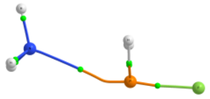

P	0.000000	0.391532	0.000000
H	-0.727788	-0.258103	1.039679
H	-0.727788	-0.258103	-1.039679
N	0.939784	-2.058675	-0.000000
H	0.301499	-2.855307	-0.000000
H	1.542485	-2.167507	0.816248
H	1.542485	-2.167507	-0.816248
F	-0.945486	1.804919	0.000000

FHSe···NH_3
Imaginary frequencies=0
SCF energy=-2556.0285414
MP2 Energy=-2556.5951233

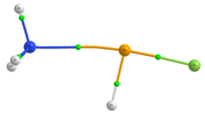

N	-2.267494	0.068744	-0.000027
H	-2.647752	0.536765	-0.823014
H	-2.648550	0.532680	0.824899
H	-2.633537	-0.883658	-0.002596
F	1.998787	0.120622	-0.000104
H	-0.028915	1.358412	0.000247
Se	0.171827	-0.091500	0.000047

FHS···NH_3
Imaginary frequencies=0
SCF energy=-553.7337665
MP2 Energy=-554.2957956

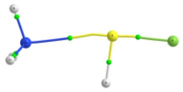

N	2.168028	0.028832	-0.000051
H	2.579981	0.476082	0.819204
H	2.578654	0.465562	-0.825614
H	2.479722	-0.942478	0.005957
F	-2.003482	0.054773	-0.000107
S	-0.293733	-0.119033	0.000099
H	-0.083492	1.210576	0.000191

H_2O···H_2O
Imaginary frequencies=0
SCF energy=-152.0874324
MP2 Energy=-152.5302069

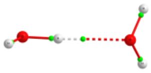

O	-1.517076	0.000160	-0.122854
H	-0.560262	0.000032	0.052907
H	-1.919859	-0.001023	0.753891
O	1.390113	-0.000125	0.112390
H	1.747813	-0.762333	-0.362396
H	1.748011	0.763045	-0.360695

H_3As···NH_3
Imaginary frequencies=0
SCF energy=-2292.1061813
MP2 Energy=-2292.4622985

N	-0.036600	2.565170	0.000000
H	0.519510	3.421112	0.000000
H	-0.648890	2.621938	0.814715
H	-0.648890	2.621938	-0.814715
H	0.940372	-0.367364	1.103166

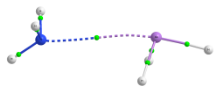

H	0.940372	-0.367364	-1.103166
H	0.361538	-2.188371	-0.000000
As	-0.036600	-0.718124	-0.000000

HCl···NH_3
Imaginary frequencies=0
SCF energy=-516.2972474
MP2 Energy=-516.6585132

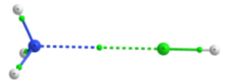

N	0.000442	2.090373	-0.000000
H	0.939904	2.488478	-0.000000
H	-0.474081	2.476806	0.816415
H	-0.474081	2.476806	-0.816415
Cl	0.000442	-1.154739	0.000000
H	-0.002340	-2.444146	0.000000

H_3P···NH_3
Imaginary frequencies=0
SCF energy=-398.6776197
MP2 Energy=-399.0222035

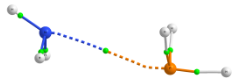

P	-0.044882	-1.179042	0.000000
H	0.874544	-0.869186	1.042540
H	0.874544	-0.869186	-1.042540
N	-0.044882	2.122510	-0.000000
H	0.280889	3.089830	-0.000000
H	-0.653024	2.025411	0.814080
H	-0.653024	2.025411	-0.814080
H	0.263482	-2.574223	0.000000

H_2S···NH_3
Imaginary frequencies=1
SCF energy=-454.9056501
MP2 Energy=-455.2649984

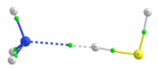

N	2.197153	0.009966	-0.000861
H	2.636068	0.770818	-0.519909
H	2.548141	-0.855146	-0.412601
H	2.577059	0.051247	0.945173
S	-1.351361	-0.082195	0.000041
H	0.005730	0.022567	-0.007102
H	-1.525287	1.255870	-0.000192

O_2NF···NH_3
Imaginary frequencies=0
SCF energy=-359.6484468
MP2 Energy=-360.6789109

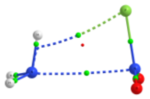

N	-0.697929	-0.227698	0.000012
F	-0.567368	1.359158	0.000059
O	-0.742330	-0.631000	-1.109829
O	-0.742230	-0.631079	1.109827
N	2.091691	-0.122671	-0.000088
H	2.684449	-0.277436	0.815810
H	1.857240	0.871446	0.000002
H	2.684768	-0.277214	-0.815798

O_2NF···H_2O
Imaginary frequencies=0
SCF energy=-379.4809554
MP2 Energy=-380.5344775

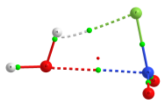

N	-0.715274	-0.172225	-0.001186
O	-0.901977	-0.460707	-1.130795
O	-0.773571	-0.638525	1.081655
H	1.785981	0.623077	0.007674
H	2.881773	-0.373624	-0.384384
O	1.993695	-0.322969	-0.010437
F	-0.245112	1.370414	0.095737

O_2NH_2As···NH_3
Imaginary frequencies=0
SCF energy=-2495.6143718
MP2 Energy=-2496.5540176

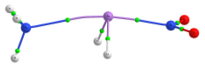

N	2.999089	0.000050	0.200924
H	3.420154	0.817465	-0.242770
H	3.420301	-0.817143	-0.243038
H	3.313890	-0.000080	1.172318
H	0.552181	1.117289	0.822138
H	0.552145	-1.117086	0.822477
N	-1.583223	0.000028	0.085569
O	-2.163682	1.099918	0.112619
O	-2.163725	-1.099840	0.112652
As	0.407562	-0.000049	-0.186022

O_2NCl···NH_3
Imaginary frequencies=1
SCF energy=-719.7403226
MP2 Energy=-720.6939934

Cl	-0.440302	0.000208	-0.000195
N	-3.232176	0.000029	-0.000010
H	-3.614215	-0.122863	0.937783
H	-3.614552	-0.751365	-0.574199
H	-3.616243	0.872761	-0.362378

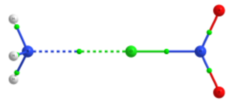	N	1.430842	-0.000051	0.000046
	O	1.933867	1.102147	0.000123
	O	1.933566	-1.102387	0.000109
$O_2NH\cdots NH_3$				
Imaginary frequencies=0	N	2.240852	-0.006342	-0.000051
SCF energy=-260.8728121	H	2.615185	0.942885	-0.002597
MP2 Energy=-261.6718456	H	2.635474	-0.476384	-0.815167
	H	2.634360	-0.471544	0.818379
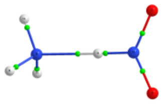	N	-0.652759	-0.001610	-0.000035
	O	-1.225401	-1.101750	0.000004
	O	-1.200781	1.110899	0.000005
	H	0.407778	-0.012485	-0.000085
$O_2NH_2P\cdots NH_3$				
Imaginary frequencies=0	P	0.486554	0.000040	-0.230937
SCF energy=-602.1812079	H	0.664584	1.056579	0.698816
MP2 Energy=-603.1070961	H	0.664850	-1.057547	0.697606
	N	3.111379	-0.000018	0.090257
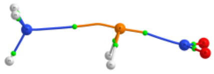	H	3.500880	0.817090	-0.381659
	H	3.500539	-0.815860	-0.384142
	H	3.498110	-0.001569	1.035317
	N	-1.378029	-0.000049	0.032105
	O	-1.953584	1.101286	0.058824
	O	-1.954006	-1.101140	0.058874
$O_2NHSe\cdots NH_3$				
Imaginary frequencies=0	N	3.017147	0.138876	-0.000136
SCF energy=-2660.6660784	H	3.394109	0.619108	-0.818195
MP2 Energy=-2661.6115725	H	3.413182	-0.801747	-0.005832
	H	3.394601	0.609452	0.823287
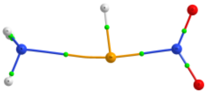	H	0.545611	1.308034	0.000107
	N	-1.564375	0.071957	0.000421
	O	-2.045804	1.208886	-0.000210
	O	-2.202006	-0.988045	-0.000294
	Se	0.384282	-0.146394	0.000078
$O_2NHS\cdots NH_3$				
Imaginary frequencies=0	N	3.168997	0.058560	0.000147
SCF energy=-658.3817621	H	3.599943	0.501958	-0.811859
MP2 Energy=-659.3223577	H	3.474519	-0.915096	-0.009062
	H	3.593369	0.484420	0.824898
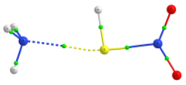	S	0.427034	-0.195992	-0.000545
	H	0.646991	1.133176	-0.001048
	N	-1.370205	0.031910	0.000096
	O	-1.822207	1.179104	0.000181
	O	-2.020156	-1.016837	0.000330
$(PH_2CN)_2$				
Imaginary frequencies=0	P	1.477940	2.013901	-0.205790
SCF energy=-868.4168976	H	0.803706	2.295348	1.014832
MP2 Energy=-869.2759021	H	2.781473	2.276825	0.314307
	C	1.582043	0.228496	0.092591
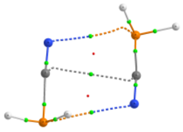	N	1.582043	-0.959869	0.171738
	P	-1.477940	-2.013901	-0.205790
	H	-0.803706	-2.295348	1.014832
	H	-2.781473	-2.276825	0.314307
	C	-1.582043	-0.228496	0.092591
	N	-1.582043	0.959869	0.171738

Table S2. Number of Electrons, EDS total values (EDS_T), EDS positive (EDS_T^+) and negative values, and errors for $H_2O\cdots H_2O$ and $NH_3\cdots HF$ bonded by hydrogen bonds at different methods and basis sets.

System	Method	Basis set	$H_2O\cdots H_2O$ (XY) ne	ne (frozen core)	ne (full density)	H_2O(X) ne	ne (frozen core)	ne (full density)	H_2O(Y) ne	ne (frozen core)	ne (full density)
$H_2O\cdots H_2O$	B3LYP	avdz	20	15.9986	20.0773	10	7.9982	10.0151	10	8.0002	10.0620
	M06-2X	avdz	20	15.9982	20.0493	10	7.9978	9.9949	10	8.0003	10.0544
	WB97XD	avdz	20	15.9976	20.0419	10	7.9980	10.0021	10	7.9995	10.0397
	MP2	avdz	20	15.9995	20.0902	10	7.9987	10.0202	10	8.0008	10.0701
		jvtz	20	15.9982	20.0608	10	7.9978	10.0014	10	8.0008	10.0598
		avtz	20	15.9987	20.0543	10	7.9980	9.9958	10	8.0009	10.0587
		avqz	20	15.9977	20.0191	10	7.9980	9.9906	10	7.9997	10.0285

System	Method	Basis set	EDS_T (XY-X-Y) FC	FD	EDS_T^+ FC	FD	EDS_T^- FC	FD	% Error FC	FD
$H_2O\cdots H_2O$	B3LYP	avdz	0.0002	0.0002	0.0769	0.0770	-0.0767	-0.0768	0.288	0.275
	M06-2X	avdz	0.0001	0.0000	0.0781	0.0781	-0.0780	-0.0781	0.135	0.029
	WB97XD	avdz	0.0001	0.0000	0.0773	0.0773	-0.0772	-0.0773	0.101	0.003
	MP2	avdz	0.0000	0.0000	0.0761	0.0762	-0.0761	-0.0762	0.029	0.016
		jvtz	-0.0004	-0.0004	0.0728	0.0729	-0.0732	-0.0733	0.527	0.560
		avtz	-0.0002	-0.0002	0.0745	0.0746	-0.0747	-0.0748	0.241	0.247
		avqz	0.0001	0.0001	0.0743	0.0744	-0.0742	-0.0743	0.288	0.275

System	Method	Basis set	$NH_3\cdots HF$ ne	ne (frozen core)	$NH_3\cdots HF$ ne	ne (frozen core)	HF ne	ne (frozen core)	EDS_T	EDS_T^+	EDS_T^-	% Error	
$NH_3\cdots HF$	M06-2X	avdz	20	15.9928	10	7.9951	10	7.9972	0.0006	0.1581	-0.1574	0.391	0.393
	MP2	avdz	20	15.9925	10	7.9950	10	7.9971	0.0004	0.1522	-0.1517	0.281	0.282
		jvtz	20	15.9928	10	7.9972	10	7.9951	0.0005	0.1528	-0.1523	0.313	0.314
		avtz	20	15.9932	10	7.9952	10	7.9973	0.0007	0.1546	-0.1539	0.463	0.465
		avqz	20	15.9933	10	7.9955	10	7.9974	0.0004	0.1540	-0.1536	0.278	0.279
		avqz	36	22.0856	26	14.0820	10	8.0014	0.0022	0.2743	-0.2721	0.391	0.393

Table S3. Input points, number of points on each direction of the grid, total number of points in the grid, increment of volume ($Bohr^3$), and electron density values (e^-) and error at the MP2/aug-cc-pVDZ for $H_2O\cdots H_2O$ system.

Input points	nx	ny	nz	grid points	dτ	EDS_T [a]	EDS_T^{+} [a]	EDS_T^{-} [a]	% Error
10	13	9	9	1053	2.1918	-0.0188	0.0582	-0.0769	32.2461
20	26	18	18	8424	0.2329	0.0034	0.0770	-0.0736	4.37538
30	39	27	26	27378	0.0655	0.0002	0.0736	-0.0734	0.26834
40	52	36	35	65520	0.0269	-0.0002	0.0740	-0.0742	0.31489
50	65	44	44	125840	0.0136	0.0000	0.0740	-0.0740	0.05643
60	78	53	52	214968	0.0078	0.0000	0.0740	-0.0740	0.01168
70	91	62	61	344162	0.0049	0.0000	0.0741	-0.0741	0.0578
80	104	71	70	516880	0.0032	-0.0001	0.0740	-0.0741	0.13536
90	117	80	78	730080	0.0023	0.0001	0.0741	-0.0740	0.07014
100	130	89	87	1006590	0.0016	0.0002	0.0742	-0.0740	0.20228
110	143	98	96	1345344	0.0012	0.0000	0.0741	-0.0740	0.04647
120	156	106	104	1719744	0.0009	0.0000	0.0740	-0.0740	0.01129
130	169	115	113	2196155	0.0007	-0.0002	0.0739	-0.0741	0.29111
140	182	124	122	2753296	0.0006	0.0002	0.0742	-0.0740	0.31468
150	195	133	130	3371550	0.0005	0.0001	0.0741	-0.0740	0.06992
160	208	142	139	4105504	0.0004	-0.0002	0.0739	-0.0741	0.23596
170	221	151	148	4938908	0.0003	0.0003	0.0743	-0.0740	0.37909
180	234	160	157	5878080	0.0003	0.0000	0.0740	-0.0740	0.04397
190	247	168	165	6846840	0.0002	-0.0003	0.0738	-0.0741	0.45421
200	260	177	174	8007480	0.0002	-0.0001	0.0740	-0.0741	0.13505

[a] those values correspond to ρ* dτ

Table S4. Intermolecular distance, electron density (ρ_{BCP}), Laplacian ($\nabla^2\rho_{BCP}$) and total energy density (H_{BCP}) at the bond critical point for all the systems studied at the MP2/aug-cc-pVDZ for $H_2O\cdots H_2O$ system.

System	Interaction	Distance	ρ_{BCP}	$\nabla^2\rho_{BCP}$	H_{BCP}
$FH\cdots NH_3$	H···N	1.689	0.051	0.139	-0.005
$O_2NH\cdots NH_3$	H···N	1.833	0.040	0.103	-0.001
$ClH\cdots NH_3$	H···N	1.726	0.051	0.107	-0.006
$F_3CH\cdots NH_3$	H···N	2.280	0.017	0.042	0.000
$H_2O\cdots H_2O$	H···O	1.951	0.024	0.086	0.002
$HSH\cdots NH_3$	H···N	2.197	0.020	0.047	0.000
$FBr\cdots NH_3$	Br···N	2.325	0.057	0.136	-0.008
$ClBr\cdots NH_3$	Br···N	2.456	0.044	0.117	-0.002
$FCl\cdots NH_3$	Cl···N	2.273	0.057	0.152	-0.005
$Br_2\cdots NH_3$	Br···N	2.517	0.039	0.108	-0.001
$Cl_2\cdots NH_3$	Cl···N	2.552	0.031	0.102	0.002
$O_2NCl\cdots NH_3$	Cl···N	2.792	0.020	0.064	0.002
$F_3CCl\cdots NH_3$	Cl···N	3.040	0.012	0.040	0.001
$HCl\cdots NH_3$	Cl···N	3.245	0.008	0.027	0.001
$FHSe\cdots NH_3$	Se···N	2.445	0.043	0.102	-0.005
$FHS\cdots NH_3$	S···N	2.466	0.037	0.098	-0.002
$O_2NHSe\cdots NH_3$	Se···N	2.648	0.030	0.074	-0.001
$O_2NHS\cdots NH_3$	S···N	2.754	0.022	0.062	0.001
$CNHSe\cdots NH_3$	Se···N	2.931	0.017	0.049	0.001
$F_3CHSe\cdots NH_3$	Se···N	3.013	0.015	0.042	0.001
$CNHS\cdots NH_3$	S···N	3.024	0.013	0.041	0.001
$F_3CHS\cdots NH_3$	S···N	3.109	0.011	0.036	0.001
$O_2NH_2As\cdots NH_3$	As···N	2.620	0.030	0.065	-0.002
$FH_2As\cdots NH_3$	As···N	2.609	0.030	0.069	-0.002
$O_2NH_2P\cdots NH_3$	P···N	2.644	0.027	0.057	-0.002
$FH_2P\cdots NH_3$	P···N	2.624	0.027	0.061	-0.002
$F_3CH_2As\cdots NH_3$	As···N	3.016	0.015	0.037	0.000
$F_3CH_2P\cdots NH_3$	P···N	3.035	0.013	0.035	0.001
$H_3As\cdots NH_3$	As···N	3.283	0.009	0.024	0.001
$H_3P\cdots NH_3$	P···N	3.302	0.008	0.021	0.001
$FNO_2\cdots NH_3$	N···N	2.792	0.013	0.050	0.002
$FNO_2\cdots H_2O$	N···N	2.713	0.011	0.055	0.002
$(PH_2CN)_2$	P···N	3.258	0.008	0.026	0.001

Table S5. LMOEDA energy components, kJ mol^{-1} for all the systems studied.

System	Electrostatic	Exchange	Repulsion	polarization	Dispersion
$FH\cdots NH_3$	-96.4	-103.4	193.8	-44.0	-8.3
$O_2NH\cdots NH_3$	-87.1	-90.1	167.9	-38.8	-1.6
$ClH\cdots NH_3$	-90.0	-143.6	261.4	-55.1	-18.3
$F_3CH\cdots NH_3$	-32.5	-32.3	56.7	-10.0	-4.7
$H_2O\cdots H_2O$	-35.7	-36.4	65.5	-9.8	-5.8
$HSH\cdots NH_3$	-28.8	-48.0	82.2	-12.8	-10.9
$FBr\cdots NH_3$	-189.3	-259.7	510.4	-111.3	-26.0
$ClBr\cdots NH_3$	-132.0	-205.1	389.1	-73.7	-29.8
$FCl\cdots NH_3$	-154.1	-235.0	455.7	-100.4	-24.1
$Br_2\cdots NH_3$	-110.2	-180.9	338.5	-61.0	-29.6
$Cl_2\cdots NH_3$	-68.5	-117.5	215.5	-37.5	-21.1
$O_2NCl\cdots NH_3$	-41.7	-59.3	104.9	-20.1	-0.4
$F_3CCl\cdots NH_3$	-21.7	-29.0	50.5	-7.3	-5.7
$HCl\cdots NH_3$	-7.8	-17.0	28.5	-3.6	-4.8
$FHSe\cdots NH_3$	-111.8	-168.2	309.0	-63.5	-23.8
$FHS\cdots NH_3$	-103.5	-159.7	292.0	-53.7	-18.0
$O_2NHSe\cdots NH_3$	-73.1	-105.6	187.9	-37.4	-11.6
$O_2NHS\cdots NH_3$	-59.0	-77.2	135.9	-25.3	-4.2
$CNHSe\cdots NH_3$	-38.5	-48.7	84.0	-13.6	-12.4
$F_3CHSe\cdots NH_3$	-28.6	-42.5	71.7	-10.6	-12.7
$CNHS\cdots NH_3$	-33.6	-38.4	66.1	-9.8	-6.9
$F_3CHS\cdots NH_3$	-26.4	-32.4	54.9	-7.8	-6.6
$O_2NH_2As\cdots NH_3$	-103.3	-142.9	259.7	-46.7	-10.0
$FH_2As\cdots NH_3$	-98.2	-145.0	262.1	-42.2	-19.9
$O_2NH_2P\cdots NH_3$	-82.3	-119.2	211.5	-38.2	-7.7
$FH_2P\cdots NH_3$	-77.4	-124.6	219.5	-35.1	-16.6
$F_3CH_2As\cdots NH_3$	-40.0	-59.1	100.3	-14.7	-9.7
$F_3CH_2P\cdots NH_3$	-32.3	-48.0	80.1	-12.0	-7.3
$H_3As\cdots NH_3$	-15.3	-30.3	49.0	-5.5	-9.0
$H_3P\cdots NH_3$	-11.3	-22.0	35.3	-3.7	-6.9
$FNO_2\cdots NH_3$	-34.8	-37.9	69.7	-7.3	-14.1
$FNO_2\cdots H_2O$	-27.2	-28.5	53.0	-7.4	-14.2
(PH_2CN)	-34.6	-55.2	92.8	-12.0	-22.4

Figure S1. Correlation between the LMOEDA and the EDS$^+$ for each family of interactions studied at the MP2/aug-cc-pVDZ computational level

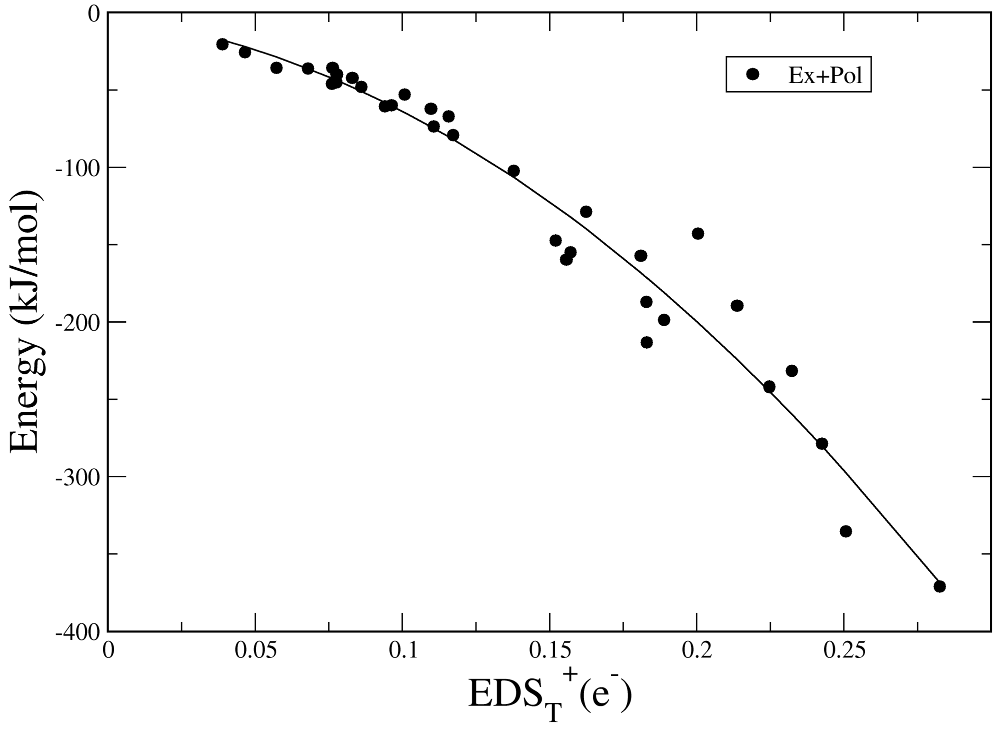

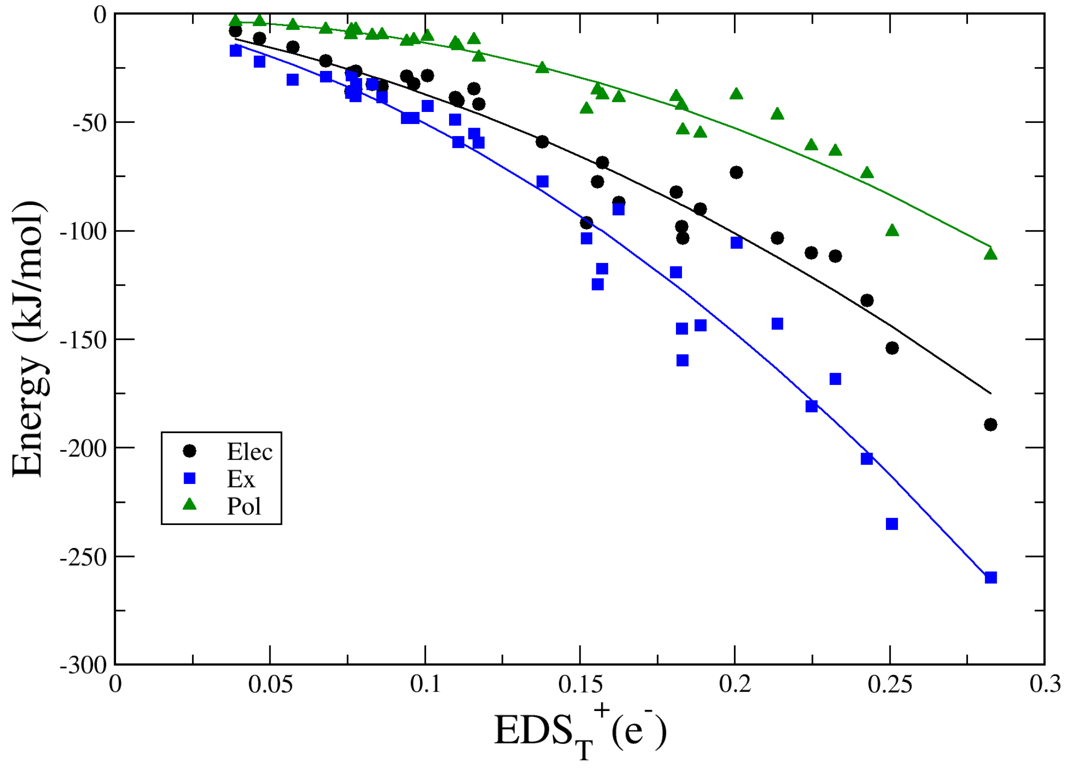

Figure S2. Evolution of the percentage of EDS inside the cut-off and EDS values (in-plots) with respect to the cut-off for FBr···NH_3 system.

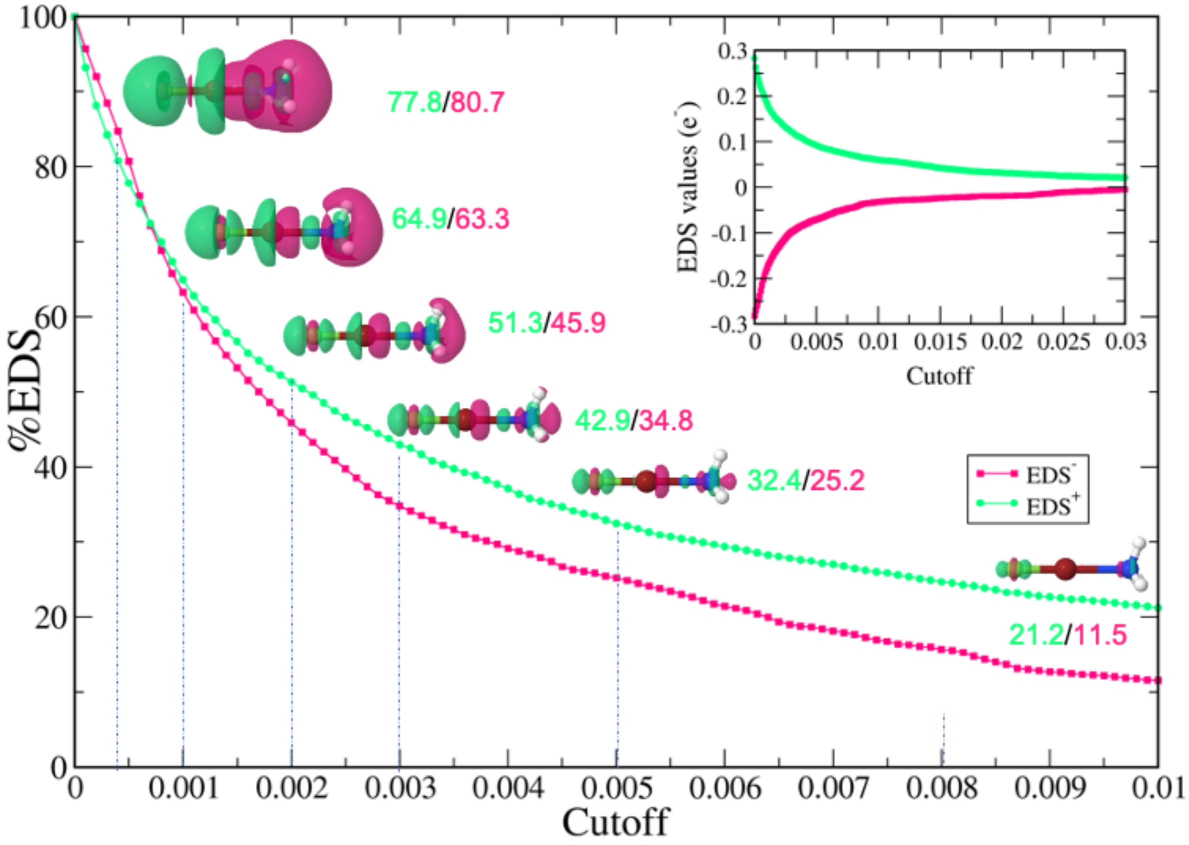

Figure S3. Evolution of the percentage of EDS inside the cut-off with respect to the cut-off for $(FNO_2)_2$ and $(ClNO_2)_2$ systems.

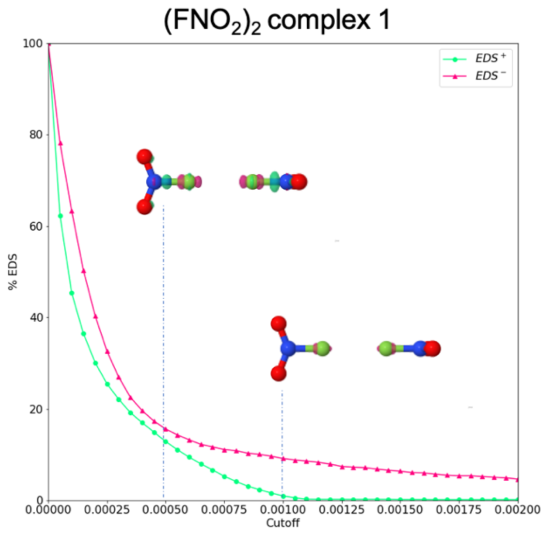

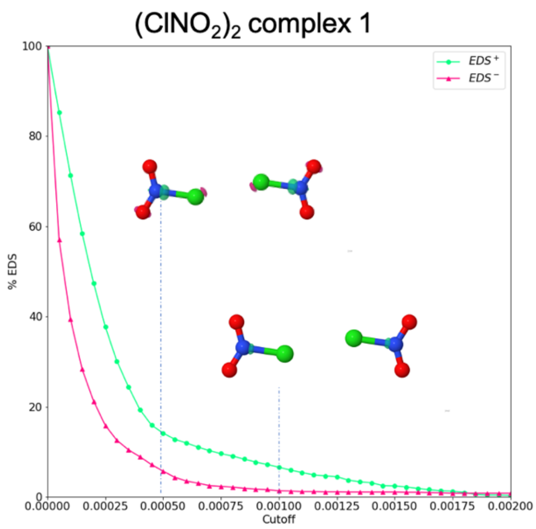

$(ClNO_2)_2$ complex 2

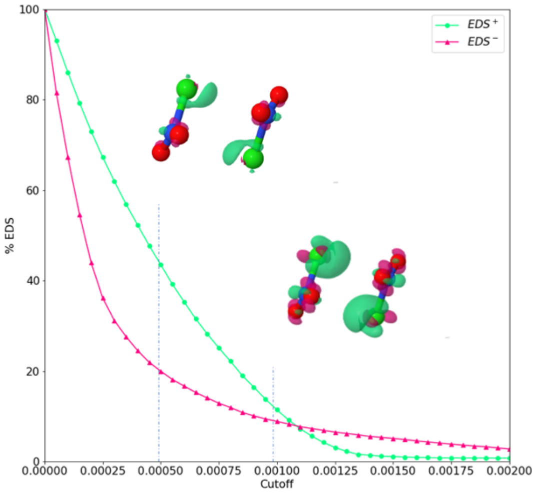

$(ClNO_2)_2$ complex 3

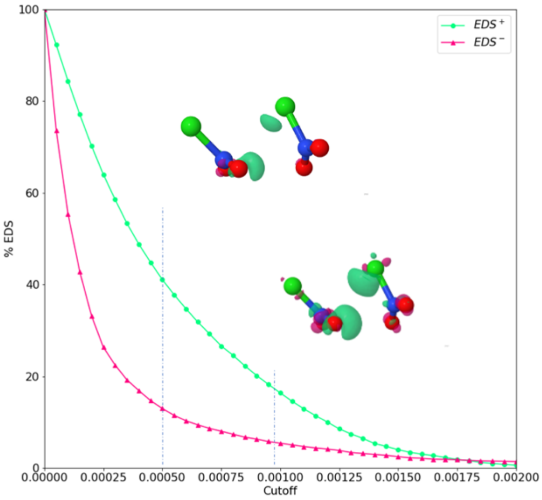

$(ClNO_2)_2$ complex 4

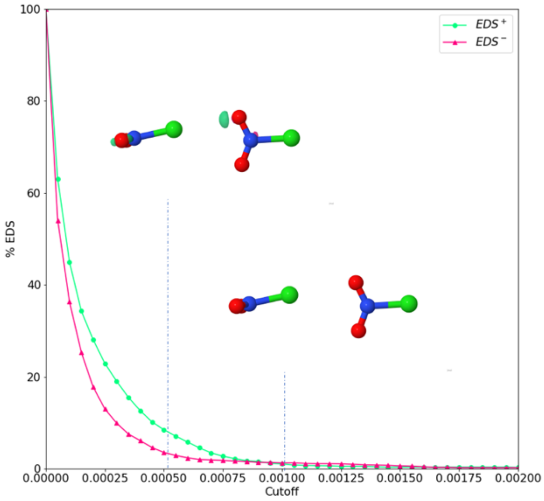

$(ClNO_2)_2$ complex 6

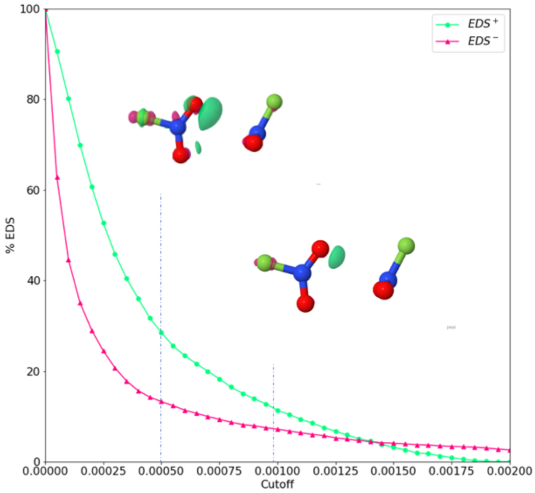

$(FNO_2)_2$ complex 5

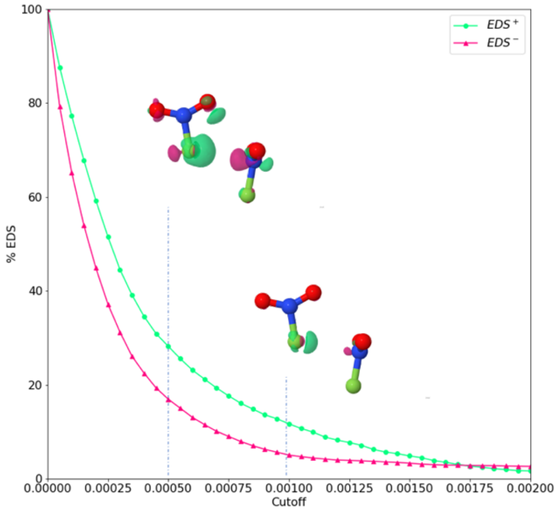

$(ClNO_2)_2$ complex 5

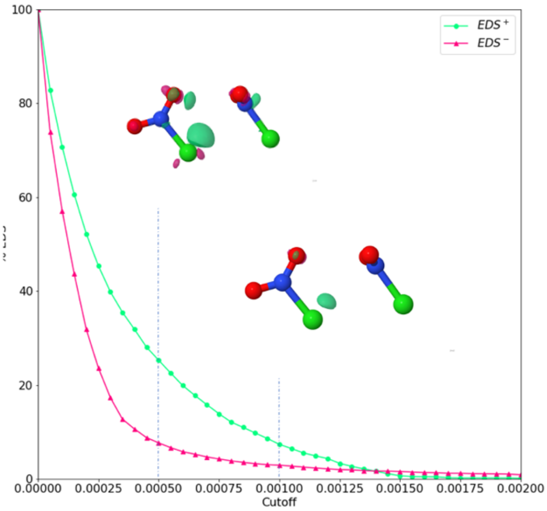

Figure S4. Electron density shift maps and EDS$^{+/-}$ values at the 0.001 a.u. isovalue for all the complexes studied at the MP2/aug-cc-pVDZ computational level.

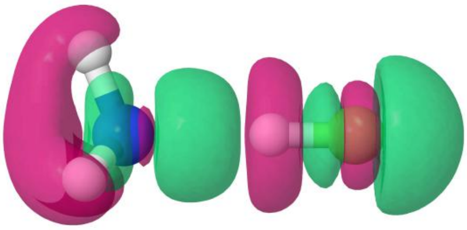

$FH\cdots NH_3$ EDS^{+}, EDS^{-}: 0.108 -0.071

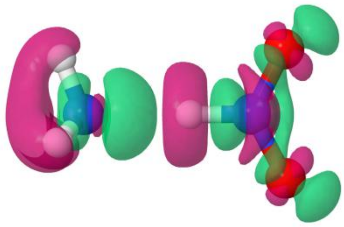

$O_2NH\cdots NH_3$ EDS^{+}, EDS^{-}: 0.088 -0.083

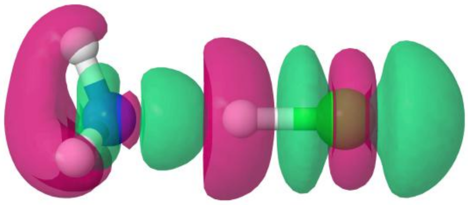

$ClH\cdots NH_3$ EDS^{+}, EDS^{-}: 0.115 -0.099

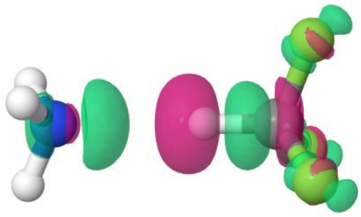

$F_3CH\cdots NH_3$ EDS^{+}, EDS^{-}: 0.036 -0.027

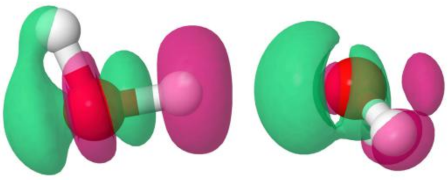

$H_2O\cdots H_2O$ EDS^{+}, EDS^{-}: 0.037 -0.028

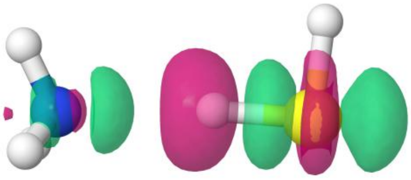

$HSH\cdots NH_3$ EDS^{+}, EDS^{-}: 0.033 -0.037

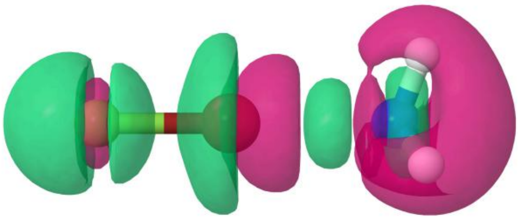

$FBr\cdots NH_3$ EDS^{+}, EDS^{-}: 0.183 -0.178

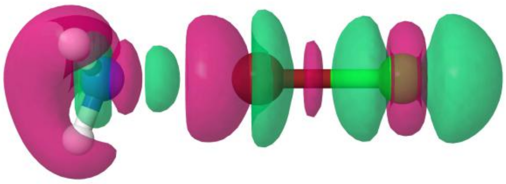

$ClBr\cdots NH_3$ EDS^{+}, EDS-: 0.140 -0.138

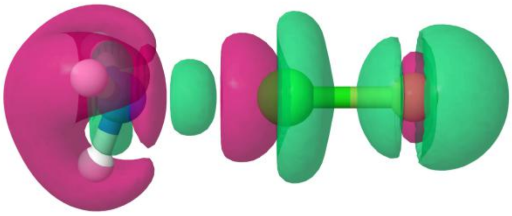

$FCl\cdots NH_3$ EDS+, EDS-: 0.165 -0.145

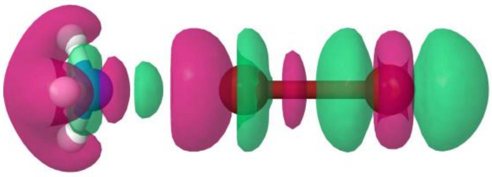

$Br_2\cdots NH_3$ EDS+, EDS-: 0.123 -0.122

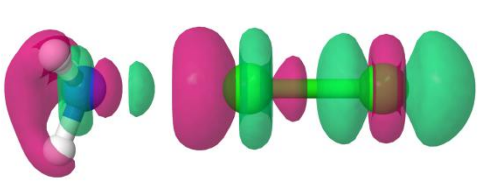

$Cl_2\cdots NH_3$ EDS+, EDS-: 0.080 -0.076

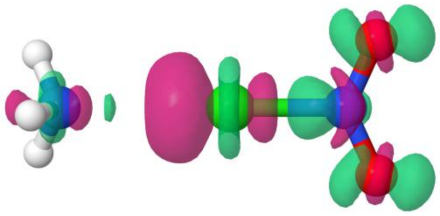

$O_2NCl\cdots NH_3$ EDS+, EDS-: 0.045 -0.049

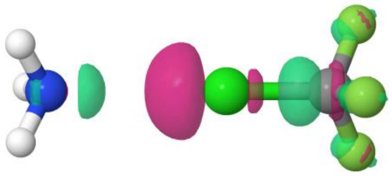

$F_3CCl\cdots NH_3$ EDS+, EDS-: 0.015 -0.021

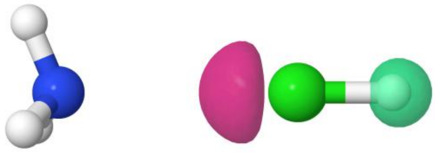

$HCl\cdots NH_3$ EDS+, EDS-: 0.005 -0.009

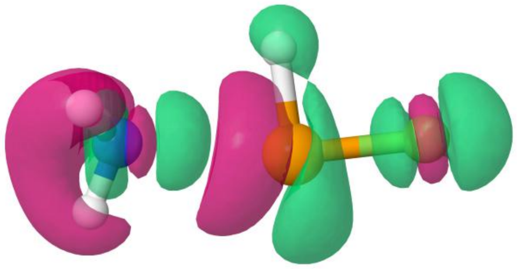

$FHSe\cdots NH_3$ EDS+, EDS-: 0.132 -0.135

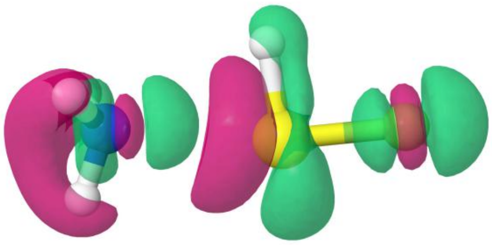

$FHS\cdots NH_3$ EDS+, EDS-: 0.099 -0.095

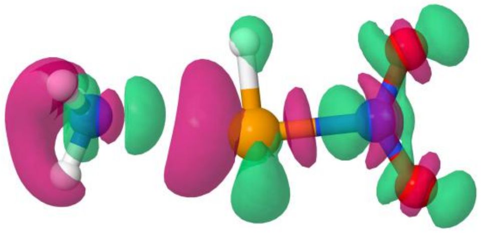

$O_2NHSe\cdots NH_3$ EDS+, EDS-: 0.094 -0.104

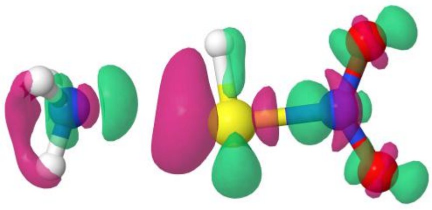

$O_2NHS\cdots NH_3$ EDS+, EDS-: 0.054 -0.058

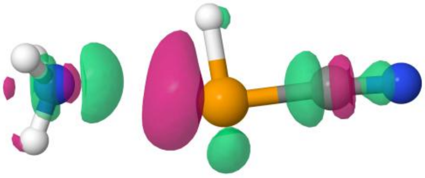

$CNHSe\cdots NH_3$ EDS+, EDS-: 0.032 -0.037

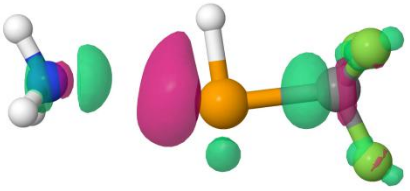

$F_3CHSe\cdots NH_3$ EDS+, EDS-: 0.030 -0.031

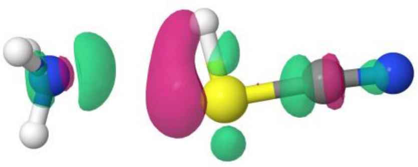

$CNHS\cdots NH_3$ EDS+, EDS-: 0.020 -0.026

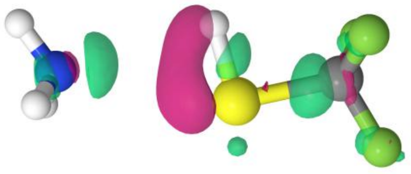

$F_3CHS\cdots NH_3$ EDS+, EDS-: 0.018 -0.020

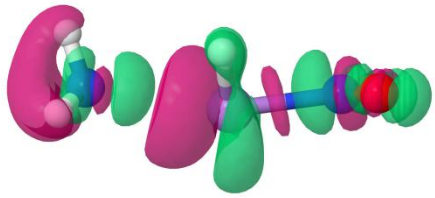

$O_2NH_2As\cdots NH_3$ EDS+, EDS-: 0.096 -0.111

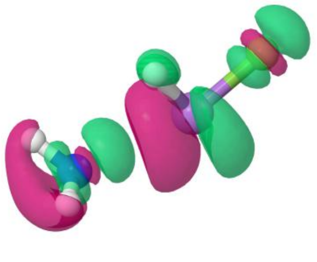

$FH_2As\cdots NH_3$ EDS+, EDS-: 0.086 -0.094

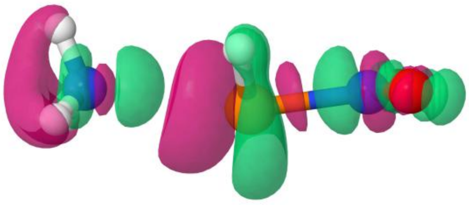

$O_2NH_2P\cdots NH_3$ EDS+, EDS-: 0.079 -0.087

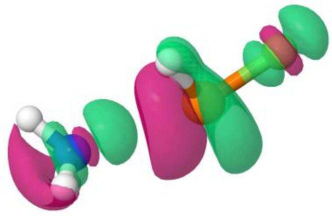

$FH_2P\cdots NH_3$ EDS+, EDS-: 0.071 -0.073

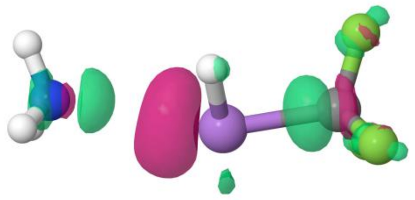

$F_3CH_2As\cdots NH_3$ EDS+, EDS-: 0.030 -0.036

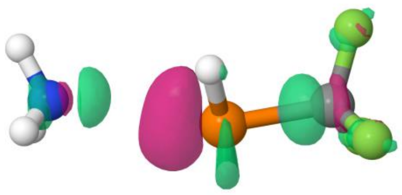

$F_3CH_2P\cdots NH_3$ EDS+, EDS-: 0.023 -0.030

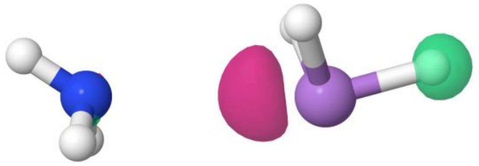

$H_3As\cdots NH_3$ EDS+, EDS-: 0.004 -0.009

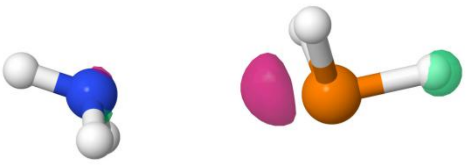

$H_3P\cdots NH_3$ EDS+, EDS-: 0.002 -0.004

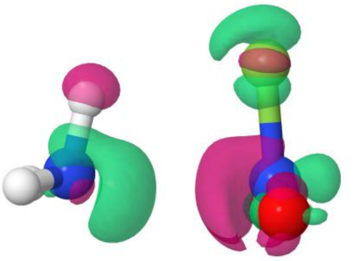

$FNO_2\cdots NH_3$ EDS+, EDS-: 0.026 -0.020

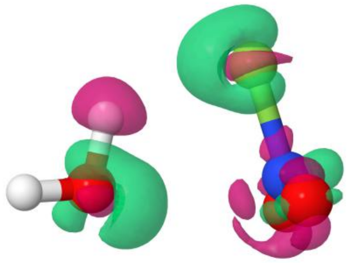

$FNO_2\cdots H_2O$ EDS+, EDS-: 0.035 -0.017

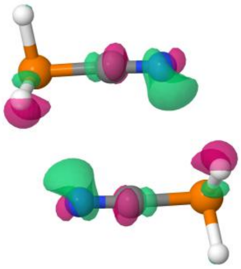

$(PH_2CN)_2$ EDS+, EDS-: 0.029 -0.020

Figure S5. Correlation between the interaction energy and the EDS$^+$ at the 0.001 au cut-off for each family of interactions studied at the MP2/aug-cc-pVDZ computational level.

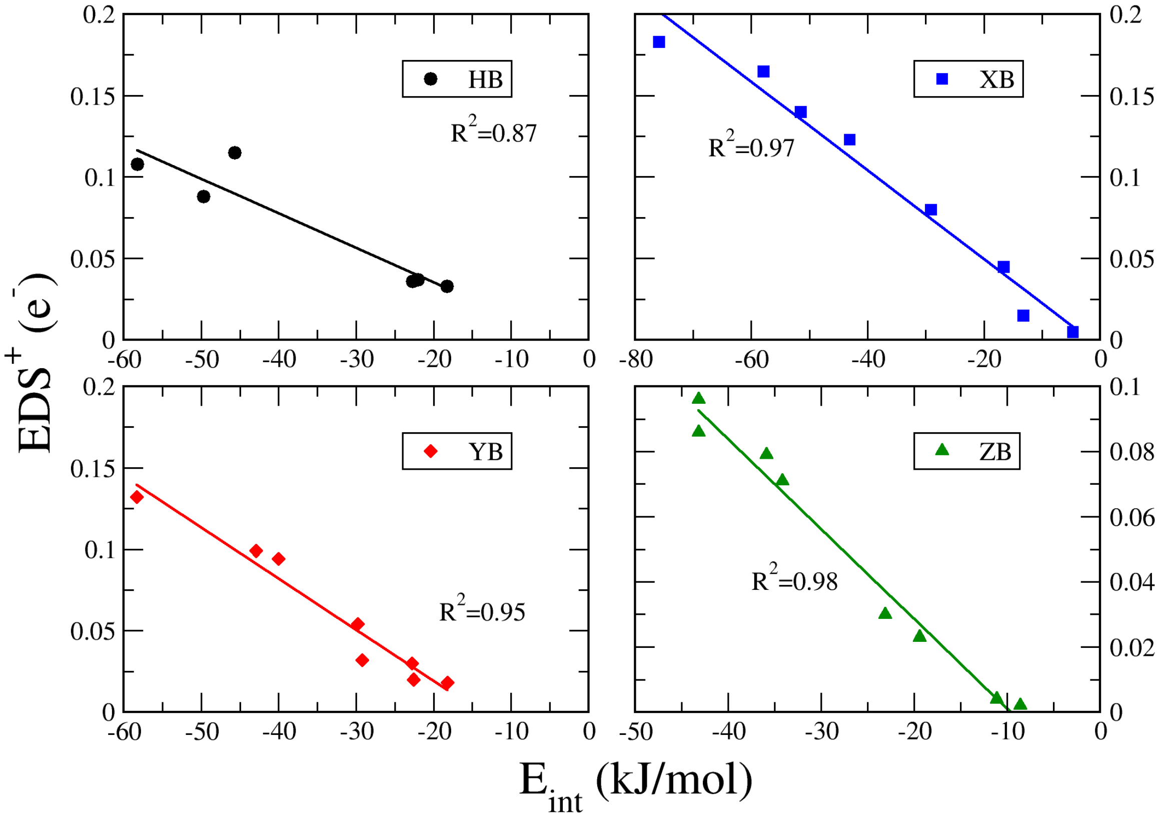

Supplement: Supplementary file 1 — jp1c00830_si_001.pdf [file jp1c00830_si_001.pdf]
